# Improving ABFE Calculations for GPCRs by Addressing Errors in Apo-State Representation

**DOI:** 10.1021/acs.jctc.6c01161

**Published:** 2026-09-28

**Authors:** Nithishwer Mouroug Anand, Antonia-Florina Panescu, Ronald M. A. Knegtel, Ewa I. Chudyk, Fabio Zuccotto, Philip C. Biggin

**Affiliations:** † Structural Bioinformatics and Computational Biochemistry, Department of Biochemistry, 6396 University of Oxford, South Parks Road, Oxford OX1 3QU, U.K.; ‡ 69292 Vertex Pharmaceuticals (Europe) Ltd, 86-88 Jubilee Avenue, Milton Park, Abingdon, Oxfordshire OX14 4RW, U.K.

## Abstract

Free energy perturbation (FEP) methods provide a robust computational framework for predicting protein-ligand binding affinities and have seen increased adoption in drug discovery workflows. Among these, absolute binding free energy (ABFE) calculations are especially valuable as they enable affinity prediction for novel compounds without prior experimental data. However, their broader adoption is limited by setup complexity, sampling challenges, and variable performance across systems. These challenges are particularly pronounced in therapeutically relevant complex membrane protein systems such as G protein-coupled receptors (GPCRs). This underscores the need to evaluate ABFE performance in GPCR systems and to develop strategies to mitigate these errors. Here, we evaluate ABFE calculations across three GPCR systems: the adenosine A_2A_ receptor (A_2A_R), the dopamine D3 receptor (D3R), and the metabotropic glutamate receptor 5 (mGlu5). We focus on three sources of systematic error in these systems: (i) inadequate resolvation of the binding pocket in apo-like alchemical states, (ii) inconsistent treatment of metal ions, and (iii) insufficient sampling of receptor conformational transitions between holo-like and apo-like ensembles. To address these issues, we introduce two methodological improvements: a biased grand-canonical-like water transport scheme to improve binding pocket resolvation, and a replica-exchange umbrella sampling (REUS)-based framework to quantify and correct the free energy associated with receptor relaxation to apo-like conformations. Together, these approaches reduce the systematic overestimation of binding affinities, improving agreement with experimental data while maintaining ranking performance. Overall, our results highlight the importance of accurately representing the apo state in ABFE calculations and provide a practical framework for applying high-accuracy free energy methods to membrane protein targets.

## Graphical abstract

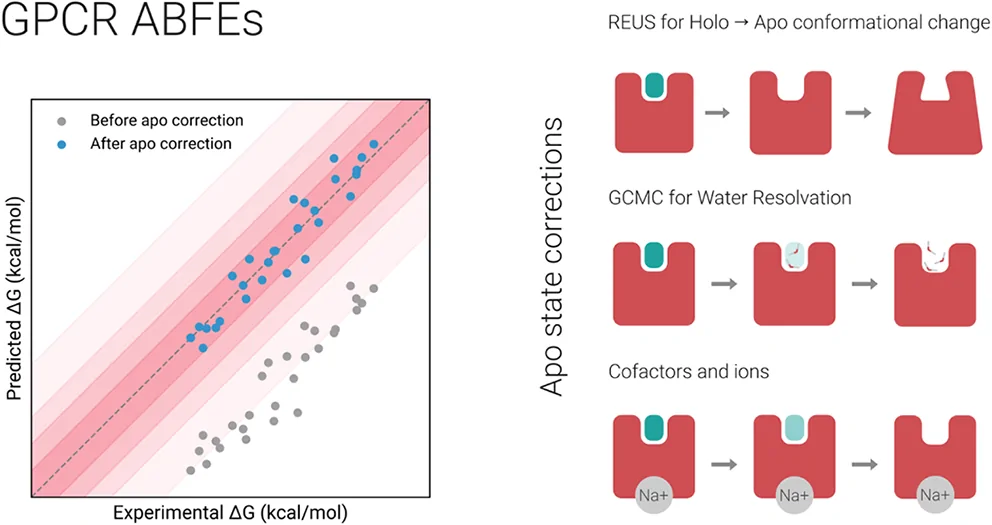

## Introduction

Reliable prediction of protein–ligand binding affinities is a critical yet unresolved challenge in computational drug discovery. Among available methods, free energy perturbation (FEP) has long been regarded as one of the most reliable approaches, owing to its rigorous thermodynamic foundation and demonstrated accuracy across diverse systems.^1,2^ FEP approaches can be broadly classified into relative and absolute binding free energy calculations (RBFE and ABFE respectively). In RBFE calculations, binding affinity differences are estimated relative to a reference ligand through an alchemical transformation between typically congeneric ligands.^3^ Because these transformations involve relatively small perturbations, RBFE calculations often benefit from favorable error cancellation and exhibit good convergence, which has led to their widespread adoption.^4^ In contrast, ABFE methods compute the absolute binding affinity of a ligand by fully decoupling it from the protein environment and comparing this to its decoupling in solution. While ABFE approaches do not require reference ligands and are therefore more generally applicable, they present significant challenges.^5–7^ The large perturbation associated with complete ligand decoupling makes convergence more difficult.^8,9^ Furthermore, ABFE calculations rely on accurate sampling of the apo state, which can involve slow protein and solvent rearrangements that are challenging to capture within accessible simulation time scales.^7,10^ These sampling challenges are particularly pronounced in membrane proteins, where slow conformational dynamics, allosteric mechanisms, and a complex lipid environment all compound the difficulty. As a result, despite their conceptual advantages, ABFE methods often exhibit lower reliability and therefore are not as widely adopted as RBFE approaches.

A systematic understanding and mitigation of the limitations of ABFE calculations is essential for their broader application in drug discovery. In this work, we therefore evaluate the performance of ABFE methods in terms of their treatment of solvation, allosteric ions and conformational change, on pharmacologically relevant systems, with a particular focus on membrane proteins. Despite their clinical importance, membrane proteins remain relatively underexplored in the context of ABFE calculations, with most prior studies focusing on soluble proteins. This is notable given that 68% of targets of FDA-approved drugs are membrane-bound or secreted^11^ (and see: https://www.proteinatlas.org/humanproteome/tissue/druggable) Among these, G protein-coupled receptors (GPCRs) represent the largest class of small-molecule drug targets, with approximately 25–36% of approved drugs targeting GPCRs. Their dynamic nature and their interactions with lipids and ions make them challenging systems for ABFE calculations.^12,13^ As important drug targets, GPCRs have been extensively studied using FEP methods, predominantly through RBFE calculations. A majority of these studies have focused on class A GPCRs as the abundance of structural data on these receptors has led to a reasonable understanding of their molecular mechanism. Class A GPCRs investigated using FEP methods include, but are not limited to, the κ-opioid receptor,^14^ orexin receptor 2,^2^ β2-adrenergic receptor,^15^ P2Y1 receptor,^15,16^ adenosine A_2A_ receptor,^13^ and adenosine A_1_ receptor.^16,17^ Among these, the A_2A_ receptor is one of the most extensively investigated systems with multiple FEP studies from different ligand series.^18^ These works have highlighted several key challenges in applying RBFE methods to GPCRs such as binding mode uncertainty, limitations in docking accuracy, stabilization or displacement of water networks, and the influence of lipid-exposed or allosteric binding sites. They also demonstrate that careful system setup and enhanced sampling strategies can mitigate these issues and yield reliable predictions.

A more systematic evaluation was provided by Guo et al.,^19^ who compiled a benchmark set for GPCR RBFE calculations spanning 12 data sets, including 131 antagonists and 8 agonists across 8 receptors (A2A, β1, CXCR4, OX2, TA1, D3, mGlu5 and δ).^13–16,20,21^ Using their in-house Uni-FEP/Hermite setup protocol, they reported an overall performance of R^2^ = 0.61 and an RMSE of 0.94 kcal mol^–1^, highlighting the current level of accuracy achievable with RBFE methods for GPCR systems.

While numerous RBFE studies have been performed for GPCRs, studies on the performance of ABFE calculations on GPCRs are scarce. In a previous study,^6^ we applied ABFE calculations to receptors with lipid-exposed binding sites, including P2Y1R and GCGR, and highlighted the impact of slow degrees of freedom, such as side-chain rotamer sampling and insufficient apo state sampling in overestimating the affinities.^22^ To address these challenges, we introduced the “apo window” approach, in which the final ten ABFE windows (where the ligand is only weakly coupled to the receptor) are initialized from the apo receptor structure to improve conformational sampling.^6^ A conceptually similar strategy was independently proposed by Liao et al., who seeded the later alchemical windows from the target receptor conformation rather than the reference, demonstrating improved sampling in the context of RBFE calculations.^22^ Other existing ABFE studies on membrane proteins, including those by Vögele et al.,^23^ focus on using ABFE-derived affinities to probe receptor function rather than rigorously evaluating their accuracy *per se*.

The limited number of ABFE studies on membrane proteins motivated us to systematically characterize their performance in these systems. Accordingly, we selected GPCR systems that have been well characterized using RBFE methods, allowing us to isolate the additional challenges introduced by ABFE. The Guo et al. benchmark data set, which includes standardized structures and RBFE results generated using a consistent protocol across all systems, offers a robust framework that minimizes protocol-dependent variability and enables identification of factors limiting ABFE performance.

To this end, we selected data sets from the receptors with the highest coefficient of determination in the DP Tech study: A2A from Lenselink et al. (R^2^ = 0.84), mGlu5 from Christopher et al. (R^2^ = 0.74), and D3 from Kumar et al. (R^2^ = 0.67). Due to compute constraints, we were unable to include all A2A data sets and excluded Piersanti et al., which had the lowest number of compounds. This yielded three receptors from the Guo et al. data set,^19^ A_2A_, D3, and mGlu5, for detailed investigation. We utilized the Guo study as the primary point of comparison because our calculations were initialized from the same structures and with identical system preparation, enabling the closest like-for-like comparison. Other studies have examined the same data sets (see SI Table S1 for a summary), but substantial variation in ligand inclusion, FEP methodology, ligand parametrization, and other system-specific parameters introduces confounding factors that complicate direct comparison.

We first established a baseline ABFE protocol using one data set and subsequently extended this approach across the selected systems. We then systematically analyzed the sources of error and explored strategies to mitigate them. On the setup side, we found that the presence or absence of bound metal ions has a variable effect on ligand affinities depending on the system. On the sampling side, we identified two key sources of error: inadequate water resolvation in the apo-like alchemical windows, and subtle conformational differences in the binding pocket from incomplete relaxation to the apo conformation. We also propose and validate methods to mitigate these problems.

## Methods

### System Preparation

The starting coordinates for the protein–ligand complex were taken from the input structures provided by Guo et al. Residue protonation states were retained as assigned in the Guo et al. structure,^19^ and structurally resolved water molecules from that model were preserved. Following best practices described by Cook et al.,^6^ the protein–ligand complex was extracted from the original coordinate set and re-embedded in a POPC bilayer using CHARMM-GUI^24^ to generate multiple alternative embeddings thereby improving sampling of interfacial lipid interactions.

The protein, lipid bilayer, and ligand were parameterized using the ff99SB-ILDN,^25^ Lipid21,^26^ and Sage force fields,^27^ respectively. Ligand parameters were generated using the OpenFF toolkit (via ParmEd/OpenMM).^28^

The membrane-embedded protein–ligand complex was subsequently solvated with TIP3P water using the GROMACS^29^ solvate module. The system was neutralized and supplemented with NaCl/KCl to reproduce the desired ionic strength using the GROMACS genion module. Where required, bound ions were introduced by structural superposition from experimentally resolved ion-bound reference structures.

### System Equilibration

The system was first subjected to a steepest-descent energy minimization using particle mesh Ewald (PME) electrostatics^30^ with a 1.2 nm real-space cutoff for both electrostatic and van der Waals interactions. Minimization was considered complete if the maximum force fell below 2000 kJ mol^–1^ nm^–1^ or after 500,000 steps.

Following minimization, a two-step equilibration procedure was followed: First, a 10 ps NVT equilibration was performed at 298.15 K using the stochastic velocity-rescaling thermostat of Bussi, Donadio, and Parrinello,^31^ implemented using the GROMACS v-rescale option, with position restraints applied to heavy atoms. Initial velocities were assigned from a Maxwell–Boltzmann distribution at 298.15 K. This was followed by a 4 ns NPT equilibration at 298.15 K and 1 bar using the stochastic velocity-rescaling thermostat and the Parrinello–Rahman barostat^32^ with semi-isotropic pressure coupling.

During equilibration, equations of motion were integrated using the leapfrog integrator with a 2 fs time step with constraints on all bonds involving hydrogen atoms using the LINCS algorithm.^33^ Temperature was controlled using the stochastic velocity-rescaling thermostat, with separate coupling groups for protein–membrane and solvent–ion subsystems. The equilibrated trajectories were then used to derive Boresch restraints^34^ using the *BoreschRestraintsGenerator* tool.^8^

### Boresch Restraints

The Boresch restraints were generated using an improved implementation of the *BoreschRestraintsGenerator* described by Alibay et al.^8^ In addition to selecting the most stable reference values for the three angles, two dihedrals, and one bond from a 100 ns equilibrium simulation, the corresponding force constants were chosen to reproduce the distributions observed during equilibrium. This approach, inspired by the restraints used in the SepTop workflow from Baumann et al.,^35^ was implemented to reduce failures of ABFE calculations arising from instabilities in the Boresch restraints.

### Absolute Binding Free Energy Calculations

Absolute binding free energy (ABFE) calculations were performed using a protocol similar to the one employed in the previous work from our lab (Cook et al.^6^).

The alchemical transformation in the complex phase was carried out in three stages: electrostatic decoupling, restraint application, and van der Waals decoupling. Electrostatic interactions were decoupled over 11 λ windows (λ = 0.0–1.0 in increments of 0.1), Boresch restraints obtained from the modified *BoreschRestraintsGenerator* described in the previous paragraph were applied over 12 λ windows (λ = 0.0, 0.01, 0.025, 0.05, 0.075, 0.1, 0.15, 0.2, 0.35, 0.5, 0.75, 1.0), and van der Waals interactions were decoupled over 21 λ windows (λ = 0.0–1.0 in increments of 0.05).

The solvent leg of the simulation was performed with the ligand in a water box, using 11 λ windows for electrostatic decoupling and 21 λ windows for van der Waals decoupling, employing the same λ schedules as in the complex phase. However, here the contribution of this restraint to the free energy was accounted for analytically.

Each alchemical window consists of a series of short equilibration steps similar to the protocol described previously for the equilibration cycles, although using 10 ps for the restrained NVT equilibration, 100 ps for the restrained NPT Berendsen barostat^36^ equilibration, and 500 ps for the unrestrained NPT Parrinello–Rahman barostat equilibration. This is followed by 20 ns of production NPT simulation. If convergence was not achieved, simulations were extended in 5 ns increments until convergence criteria (described below) were satisfied.

A total of three independent replicas of the ABFE cycle are simulated for each protein–ligand pair. For the protein–ligand complexes, each replica is independently equilibrated and uses different orientational restraints, allowing the replicas to more representatively sample the available conformational space within the binding site. In selected cases (e.g., the Lenselink et al. data set^13^), additional replicas were performed during workflow optimization. All ABFE simulations were carried out using GROMACS 2020.6 ^29^ in combination with PLUMED, employing Hamiltonian replica exchange between alchemical windows, with exchange attempts every 200 MD steps (0.4 ps) unless otherwise specified. For charged ligands, a coannihilation scheme with an alchemical counterion was used to maintain system neutrality throughout the transformation. ∂H/∂λ data for all simulations have been deposited in Zenodo and are publicly available.

### Analysis of FEP Results

The ∂H/∂λ files obtained were processed using the ABFE workflow available within the Alchemlyb^37^ library. For each alchemical window, the first 1 ns of simulation was discarded as equilibration. The remaining samples were decorrelated based on the statistical inefficiency of the ∂*H*/∂λ time series. Convergence was assessed by comparing the difference between the affinity estimates using the first and second halves of the ∂*H*/∂λ data. Simulations were considered converged when the difference between these estimates was less than 0.5 kcal mol^–1^.

Uncertainties were estimated as the standard deviation of the free energy across independent replicas. All other trajectory analyses were performed using PENSA,^38^ MDAnalysis,^39^ NumPy, SciPy, and scikit-learn, while plotting was carried out using Matplotlib. Molecular visualizations were generated using ChimeraX^40^ and PyMOL.^41^ Agreement between calculated and experimental binding affinities was assessed using Spearman’s ρ, Pearson’s R, mean unsigned error (MUE), and root-mean-square error (RMSE).

### Resolvating with Biased-GCMC Simulations

To resolvate the binding pocket in apo-like windows of the ABFE simulations, we employed a solvation strategy that explicitly accounts for the alchemical state of the ligand. This approach allows a larger number of water molecules to occupy the binding pocket in windows where the ligand is weakly coupled, and fewer waters where the ligand is strongly coupled, thereby enhancing sampling efficiency and convergence across the alchemical pathway.

We implemented this strategy using a modified version of the *Grand* package developed by the Essex laboratory,^42^ extended to support a perturbed ligand that is partially coupled to the environment. To perform simulations in OpenMM^43^ using the Grand package, the system was converted from GROMACS to AMBER format using *tleap*, *ParmEd*, and the OpenFF toolkit, and subsequently imported via the generated prmtop and inpcrd files. Solvation simulations were then carried out within this OpenMM representation using the modified Grand framework. For each alchemical window, simulations were initiated from the initial ligand pose with the appropriate coupling parameters and Boresch restraints applied. Water exchange was performed using a biased grand-canonical-like Monte Carlo water transport scheme. The Adams B parameter was left at its default setting in GRAND (adams = None) and was calculated internally from the water excess chemical potential and GCMC sampling volume. In this approach, the local chemical potential of water within the binding pocket was equilibrated via Monte Carlo insertion and deletion moves. However, to preserve a fixed total number of atoms required for Hamiltonian replica exchange compatibility with windows lacking additional waters, water molecules were not created or destroyed. Instead, waters were transported between the bulk solvent and the binding pocket. As such, this method constitutes a biased grand-canonical-like water transport scheme rather than a true grand canonical Monte Carlo simulation. A graphical representation of this process is illustrated in SI Figure S1.

Each window was subjected to an initial equilibration simulation for water resolvation and the maximally solvated structure obtained from this simulation was used for the FEP simulation. The GCMC-like simulation consisted of 10,000 Monte Carlo water-transport moves over 5 ps (200 moves every 100 fs), followed by 5,000 moves over 50 ps (100 moves every 1 ps of MD). From the resulting trajectory, we selected the configuration containing the maximum number of water molecules within the predefined binding pocket region – A GCMC sphere of 4 Å radius, centered at the center of mass of the Cα atoms of residues lining the subpocket: Pro655^3.40^ and W785^6.48^ as this coincided with the center of the ligand. This maximally solvated configuration was then used as the starting structure for the corresponding apo-like ABFE window.

From the perspective of the ABFE calculation, this procedure serves as an enhanced sampling step that preferentially initializes apo-like states with fully solvated binding pockets, thereby reducing hysteresis and improving convergence of the free energy estimates. The modified grand package is available at: https://github.com/Nithishwer/grand/tree/alchemical_ligand

### Apo State Correction: Binding Pocket Characterization and CV Design

The binding pocket was characterized using a protocol inspired by Vögele et al.^23^ We featurized each simulation frame by computing pairwise Cα–Cα distances between all residues located within 6 Å of any ligand atom. These distances were used as structural descriptors of the binding pocket. Dimensionality reduction was performed using principal component analysis (PCA) on these features. We initially explored nonlinear and nondeterministic methods such as Uniform Manifold Approximation and Projection (UMAP) and t-distributed Stochastic Neighbor Embedding (t-SNE), which provided clear separation between conformational ensembles. However, PCA was ultimately selected due to its deterministic nature and the orthogonality of its principal components, which makes them a suitable collective variable for umbrella sampling. Since the start of this work, neural network–based feature extraction methods have emerged and may offer improved representations, which could be explored in future studies.^44^ Projection onto the first two principal components (PC1 and PC2) enabled clear separation of conformational ensembles corresponding to apo simulations, holo structures, and alchemical ABFE windows.

Although this representation provided a useful low-dimensional visualization of the conformational landscape, performing umbrella sampling directly in the two-dimensional PC1–PC2 space would be computationally expensive and difficult to converge. We therefore constructed a one-dimensional collective variable that captures the dominant structural differences between apo-like and holo-like conformations.

To construct this collective variable, frames were first projected onto the principal component space, standardized to zero mean and unit variance, and clustered using K-means (k = N) to identify distinct conformational states. An apo-like cluster was then selected based on its overlap with the apo simulation ensemble. In cases where multiple apo-like clusters were identified, the ones with the highest population of apo structures were used. Frames belonging to the apo cluster and to the most apo-like (vdw.20) window of the ABFE ensemble were then extracted for further analysis. From the set of all features, the subset of features that exhibited the largest differences between the apo and ABFE ensembles was identified (top 200 features based on intercluster variance). PCA was then applied to this reduced feature set using the combined subset of apo and ABFE frames. The first principal component obtained from this analysis captures the dominant structural variation between apo-like and ABFE conformations and hence was suitable to be used as a one-dimensional collective variable. This CV facilitates efficient sampling of the free energy landscape associated with transitions between conformational states of interest in subsequent umbrella sampling simulations.

### Umbrella Sampling Simulations for Apo State Correction

The collective variable (CV) described above was then used to perform umbrella sampling to characterize the free energy landscape associated with transitions between conformational states. Initial structures were selected from the centroids of clusters identified along the CV. Intermediate configurations between these states were generated using MEMENTO,^45^ which employs template-based structure modeling to produce physically reasonable protein conformations from interpolated coordinates. The MEMENTO membrane protein workflow was followed to generate 24 intermediate structures spanning the transition.

We then performed equilibration simulations on each of the interpolated structures using a standard protocol consisting of energy minimization, followed by NVT and NPT equilibration stages. This was followed by a 10 ns restrained simulation with harmonic restraints with a force constant of 1000 kJ mol^–1^ nm^–2^ applied to Cα atoms to allow relaxation of side chains while maintaining the overall backbone conformation.

The equilibrated structures were then used as initial configurations for replica exchange umbrella sampling (REUS), implemented using PLUMED,^46^ with the PCA-derived CV described above. Each umbrella window was simulated for 20 ns, with replica exchange attempts performed every 200 steps. In cases where convergence was not achieved, simulations were extended in increments of 20 ns until satisfactory convergence was obtained.

Convergence was assessed by comparing free energy estimates obtained from first and final halves of the sampled collective variable, with convergence defined as agreement within 2 kcal mol ^–1^.

## Results

### Benchmarking ABFE Protocol Sensitivity to Sampling Strategies in the Lenselink et al. A2A Data Set

We first evaluated Absolute Binding Free Energy (ABFE) calculations on membrane protein systems well-characterized by relative binding free energies (RBFE). This approach minimizes errors from binding conformations and force fields, while allowing a direct comparison with RBFEs. Using a system with known RBFE performance also helps isolate ABFE-specific errors, such as assumptions about the apo-state conformation. For our initial study, we selected the Lenselink et al.^13^ adenosine A_2A_ receptor (A_2A_) data set, where in a recent study^19^ RBFE calculations captured the maximal experimental variance (compared to 12 other GPCR systems) with an R^2^ of 0.84. We first assessed the performance of four different sampling strategies on this data set to establish the likely best protocol for ABFE calculations going forward.

For our baseline protocol, starting structures and Boresch^47^ restraints were derived from three independent 100 ns equilibration runs. For each structure, three independent ABFE repeats were performed, resulting in nine ABFE estimates per compound across seven compounds. Comparison with experimental data from Lenselink et al. revealed that variability between ABFE estimates originating from the same Boresch-restrained structure was relatively small (see Figure S2). In contrast, the variability between estimates obtained from different Boresch-restrained structures was typically larger (Figure S2). This increased spread between distinct initial structures indicates that systematic differences associated with the choice of Boresch restraint and starting structure contribute more to ABFE uncertainty than repeat-to-repeat noise within a given setup. This higher error between different initial structures reinforces the existing best practice of using multiple different decorrelated initial structures^8,48^ to reduce error and improve sampling. When ABFE estimates were averaged across the three starting structures derived from independent equilibration simulations, agreement with experiment was good, yielding an RMSE of 1.59 kcal/mol, a Spearman ρ of 0.71, and a Pearson R of 0.74 (Figure 1a).

**Figure 1:**
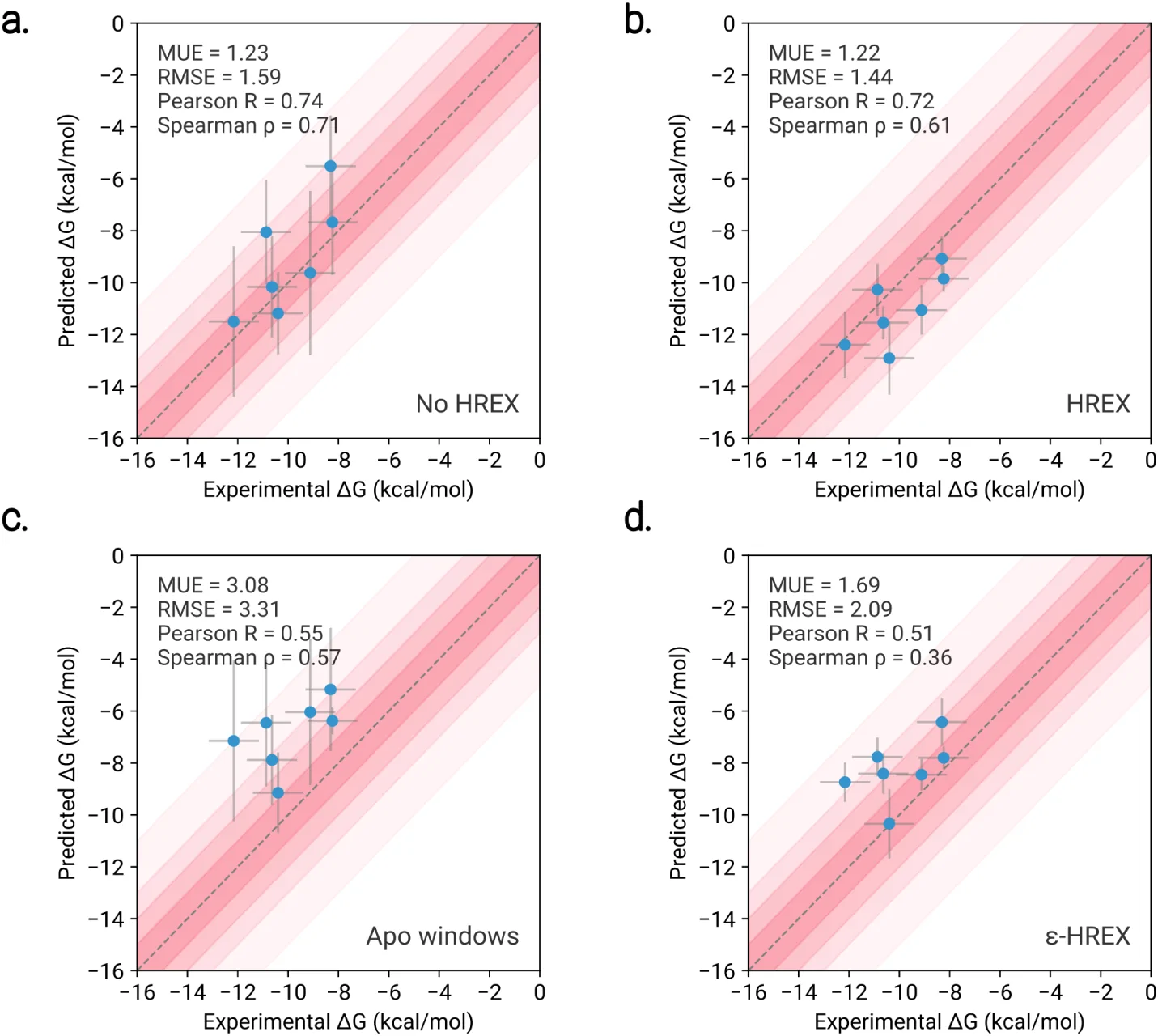
Effect of different sampling techniques on the performance of ABFE calculations for the A2A data set from Lenselink et al.^13^ (a) ABFE calculations performed without HREX. (b) ABFE calculations with HREX. (c) ABFE calculations with the final 10 λ windows initialized from apo-state structures. (d) ABFE calculations performed with ε-HREX applied. In panel (a), three ABFE replicates were performed for each of three Boresch-restrained structures (3 × 3). In panels (b–d), three ABFE replicates were performed, each initiated from an independent equilibration simulation. In all panels, shaded pink bands indicate absolute deviations from experimental binding free energies of 1, 2, 3, and 5 kcal mol–1. Experimental reference values are shown with an assumed uncertainty of ±1 kcal mol–1. Plotted values correspond to the mean binding free energy across replicate simulations, with error bars representing the standard deviation.

Having shown that statistical averaging reduces initial bias, we explored the influence of three techniques that should enhance sampling: HREX (Hamiltonian Replica Exchange) (Figure 1b), ABFEs with apo-state corrections (Figure 1c)^6,22^ and ε-HREX (Figure 1d), where the ligand’s LJ parameter, epsilon was scaled to enhance sampling.^10^ ε-HREX was first introduced by Baumann et al.^35^ to increase sampling in FEP calculations using the separated topology method. HREX performed best, with average standard deviation between interstructure ABFEs decreasing significantly (2.4 kcal mol^–1^ to 1 kcal mol^–1^) (Figure 1b), highlighting the benefit of exchanges between windows. A naïve Apo-state correction,^6^ whereby the final 10 windows of the annihilation step are seeded from an apo state model, rather than the holo-state, did not improve the accuracy (Figure 1c). This underscores the need for a more nuanced treatment of the apo state (see later). Naively initializing late λ windows from apo structures, especially without replica exchange, can shift the system toward an apo-like ensemble and lead to underestimated free energies. ε-HREX (Figure 1d) generally underestimated affinities, likely due to smoother epsilon scaling exploring unfavorable conformations. A complete breakdown of all individual compounds is given in SI Table S2. Based on these results, we adopted a protocol with HREX ABFEs from three starting frames obtained from three independent 100 ns equilibration simulations and used this as the default for the remainder of the paper.

### Binding Pocket Resolvation as a Source of Systematic Error in ABFE Calculations of the mGlu5 Receptor

We next investigated the mGlu5 data set from Guo et al., comprising binding affinities for twelve negative allosteric modulators (NAMs) characterized by Christopher et al.^49^ (Figure 2a). This system is very different from the A_2A_ system, as Class C GPCRs such as mGlu5 exhibit a markedly different transmembrane architecture, leading to a more occluded and deeply buried binding cavity (Figure 2b,c).

**Figure 2:**
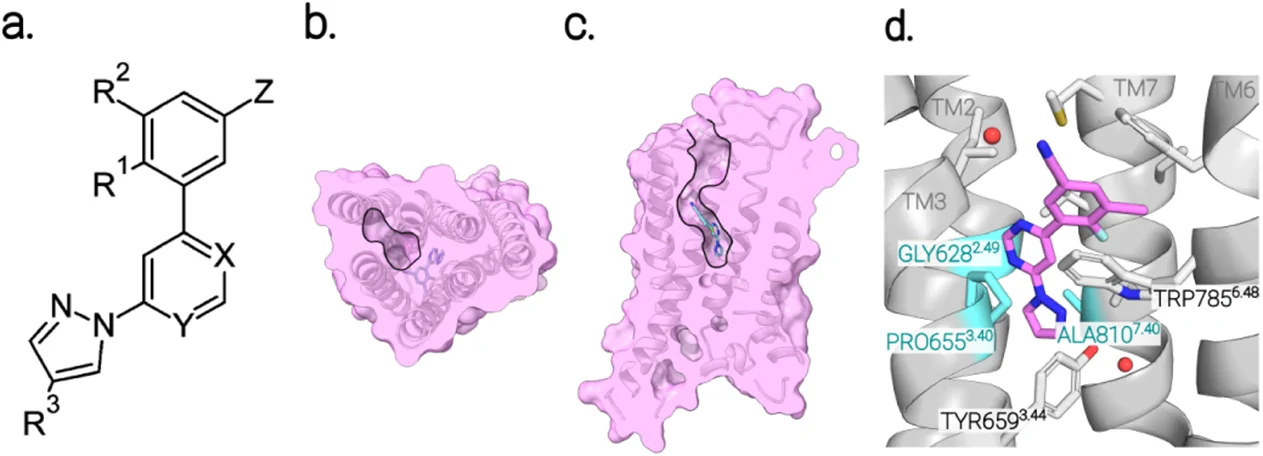
mGlu_5_ receptor binding pocket with compound 14. (a) Chemical scaffold of compound 14. (b) Cross-sectional top view of the receptor cavity, illustrating the narrowness of the binding pocket. (c) Cross-sectional side view highlighting the depth of the cavity. (d) Binding pocket of mGlu_5_ with compound 14 bound, with key interacting residues highlighted.

In mGlu5, the NAM binding site is deep and features a narrow subpocket made accessible by small side chains (Figure 2d) (Gly628^2.49^, Pro655^3.40^, Ala810^7.41^) that leave additional space at the bottom of the cavity. This subpocket is fully occupied by the distal moieties of mGlu5 NAMs, including mavoglurant, basimglurant, and the SAR-series compounds reported by Christopher et al. Because these ligands engage the same tightly confined pocket and adopt highly similar binding poses, the RBFE calculations perform well, likely benefiting from substantial cancellation of systematic errors across the series.

It is worth noting that the structure used from Christopher et al. (PDB: 5CGC) had several structural water molecules resolved, which were retained in Guo et al. data set and hence in our input structures. Two of these waters are closer to the ligand (shown in Figure 2d): one sits within the subpocket and mediates ligand interaction with Tyr659^3.44^ and Thr781^6.46^ while the other is closer to the extracellular region, mediating interactions with Val740^5.40^.

We expected the narrow, deep pocket to cause sampling issues, since its small diameter (6 Å at the narrowest point) limits solvent access to the binding pocket on typical simulation time scales. Therefore, this system provides a useful test case for probing resolvation of the binding pocket in λ windows approaching the apo state. Inadequate sampling of the resolvation could under-stabilize apo-like windows, leading to systematically overestimated binding affinities. Our preliminary FEP results supported our concerns.

Our initial FEP study, using the ligands from Christopher et al.,^49^ yielded an RMSE of 8.31 kcal mol^–1^, a Pearson’s R of 0.43, and a Spearman’s ρ of 0.57 (Figure 3a). Although the relative ranking of most compounds was captured reasonably well (with three outliers), the absolute binding free energies were severely overestimated by an average of 7.5 kcal/mol (Figure 3a). To understand this, we examined the solvation of binding pockets across ABFE windows (Figure 3b). Most compounds showed some resolvation during annihilation, but almost none reached the apo-level solvation in the fully annihilated window, as defined by three 1-μs apo mGlu5 simulations (gray lines, Figure 3b).

**Figure 3:**
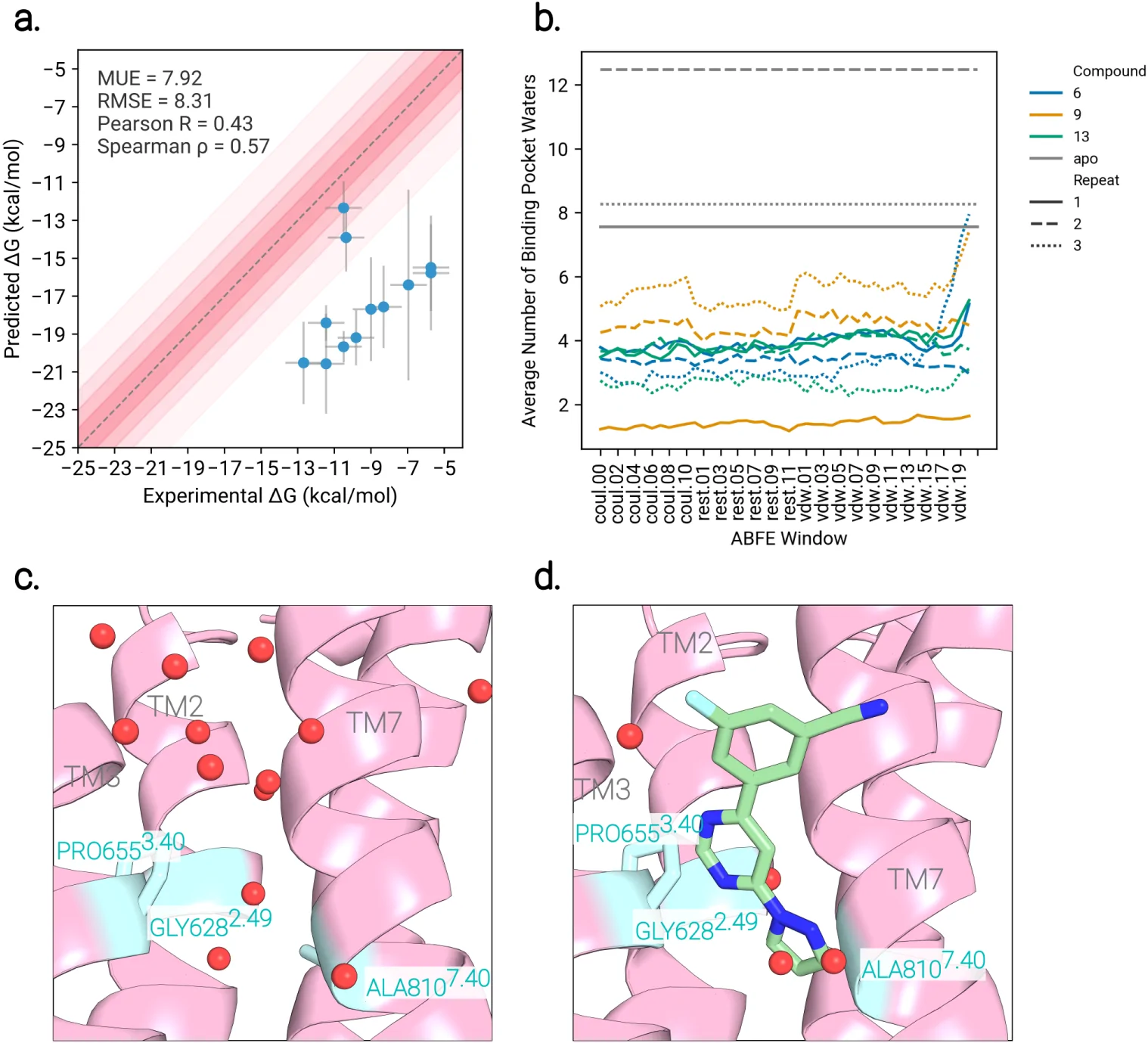
Solvation of mGlu5 in ABFE calculations. (a) Predicted vs experimental ABFE performance for the Christopher et al.^49^ mGlu5 ligand data set. (b) Water occupancy (defined as number of waters within 10 Å of the binding pocket center of mass) in each ABFE window for 3 of the compounds (6, blue; 9, orange and 13, green) compared to apo mGlu5 simulations (gray lines). (c) Cartoon representation of the binding pocket occupied by water molecules (spheres) in the apo state (13 waters in this snapshot according to the criterion described in b). (d) Corresponding cartoon showing the fully annihilated ABFE window of Compound 6, showing only four water molecules within the pocket; the ligand is present in a noninteracting state. Key residues of the subpocket are highlighted in cyan in both (c) and (d) (as in Figure 2).

Exceptions were observed for compounds 12, 15, and 17, where apo-like solvation was sampled in some repeats. In these cases, particularly for compounds 15 and 17, the predicted binding affinities were closer to the experimental values (SI Table S3), although they remained overestimated. To address this issue, we sought to enhance water resolvation in the ABFE annihilation windows by initializing the calculations from a more highly solvated binding-pocket configuration. Similar approaches, in which calculations are initialized from alternative starting structures to enhance sampling of the apo-like state, have been reported in previous FEP studies.^6,22^ We implemented a biased Grand Canonical like transport scheme that transfers water molecules from the bulk reservoir into a predefined binding site to equilibrate the local chemical potential (SI Figure S1). We then selected the most highly solvated frame from the trajectory and used it as the starting structure for all van der Waals windows (the latter half of the ABFE window sequence), in which the ligand progressively decouples from its environment.

Figure 4a shows that using the biased Grand Canonical like transport scheme reduced the RMSE from 8.31 to 4.89 kcal mol^–1^ while maintaining ranking performance (Spearman ρ = 0.57). A full compound-by-compound breakdown is given in SI Table S3. Water-occupancy plots (Figure 4b, SI Figure S3) indicate that most compounds now reach apo-level solvation in the final van der Waals leg, demonstrating the scheme’s effectiveness in introducing water into the binding pocket. Similar observations were made for the Lenselink et al. A2A system studied in the previous section (SI Figure S4) and indeed for all other data sets studied in this work.

**Figure 4:**
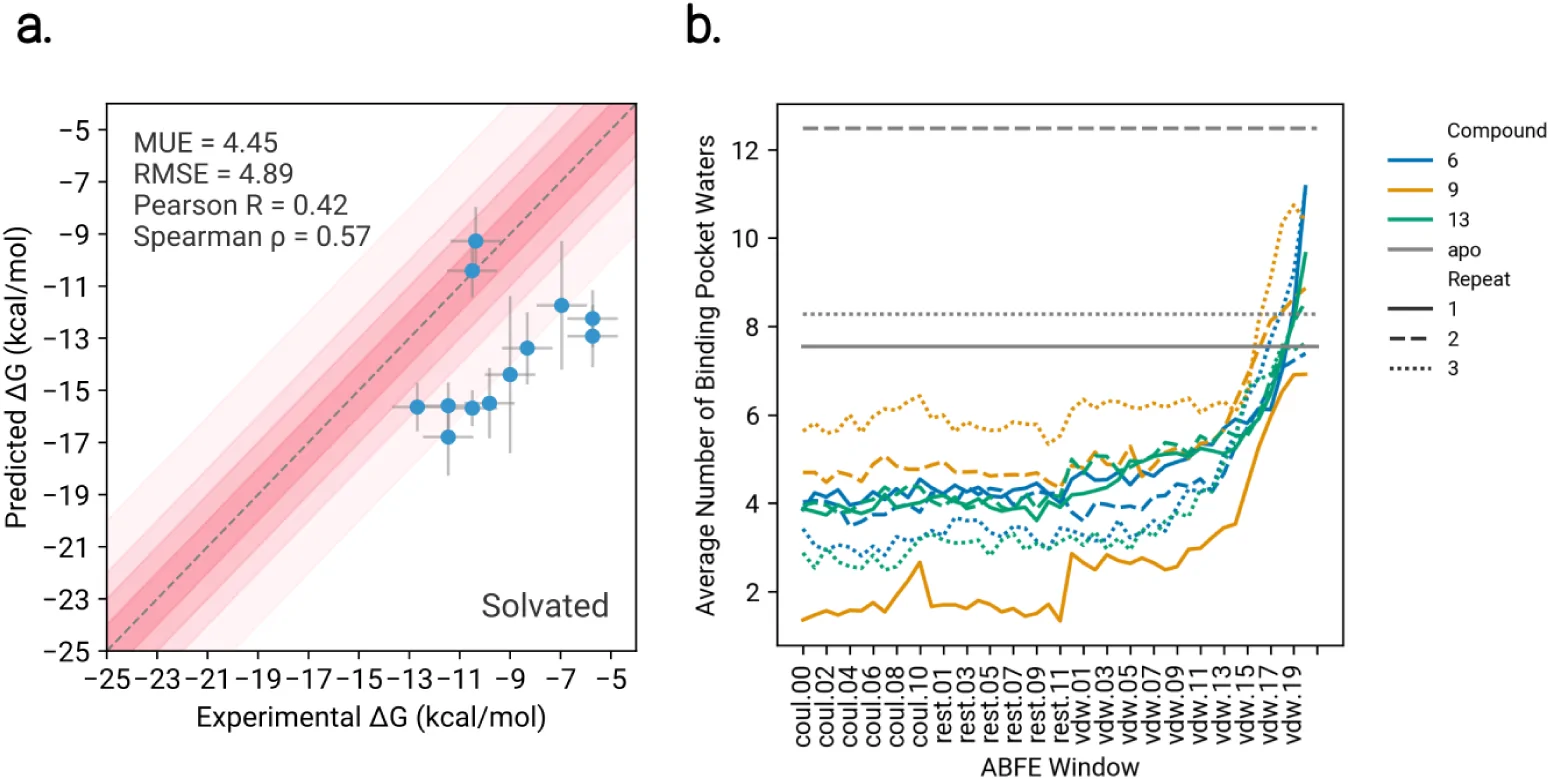
Improved performance of ABFE calculations due to enhanced resolvation of apo-like states. (a) Performance of ABFEs in the mGlu5 data set and (b) Solvation level of each of the ABFE windows across select compounds compared to apo simulations.

We still observe a significant overestimation of approximately 4 kcal mol^–1^, which may reflect allosteric modulatory effects arising from the extracellular Venus flytrap domains, not included in the present simulations but known to play an important role in Class C GPCR function and present in the experimental assay. Nonetheless, the biased GCMC transport scheme substantially reduces the magnitude of the overestimation while preserving the ability to rank compounds accurately.

Given that implementation of this protocol should not hamper performance in systems where solvation is not likely to be an issue, we tested the GCMC approach on the Lenselink et al. A2A data set. As expected, the performance is extremely similar to the HREX standard approach (SI Figure S5).

### Allosteric Sodium as a Source of Systematic Bias in A2A ABFE Calculations

Biologically abundant metal ions such as Na^+^, Mg^2+^, Ca^2+^ and Zn^2+^ have been known to modulate GPCR activity by stabilizing the receptor’s inactive state and thereby preventing receptor activation. Among these, Na^+^ has been extensively characterized as an endogenous negative allosteric modulator for many class A GPCRs.^50^ The allosteric Na^+^ typically occupies a binding pocket within the transmembrane domain, located near the highly conserved Asp^2,50^ and is known to form a coordination network involving surrounding polar residues and structured water molecules.^50^

Structural studies of the A_2A_ adenosine receptor have resolved a Na^+^ binding site^51^ that coordinates residues such as Ser^3.39^ and Asp^2.50^. In contrast, for some receptors such as the dopamine D3 Receptor, although sodium has been shown to modulate ligand binding, there is no direct experimental resolution of the ion in available structures. For other receptors like mGlu5, the role of transmembrane ion binding in modulating receptor activity remains largely unexplored.

As a consequence of the uneven experimental characterization of ion binding sites, the treatment of ions during system preparation for free-energy perturbation (FEP) calculations often varies across studies. Some computational protocols explicitly include ions in the binding pocket^2,13^ whereas others omit them,^21^ particularly when a reasonable agreement with experimental data is obtained without their inclusion. This variability is evident in the calculations performed by Guo et al. While the A_2A_ receptor systems with ligands from Minetti, Piersanti and Deflorian explicitly included the Na^+^ ion, those from Lenselink et al. did not include them. Notably, despite the absence of the sodium, the Lenselink data set exhibited the highest correlation with experimental binding affinities (R^2^ = 0.84) among all data sets analyzed by Guo et al. Motivated by this apparent inconsistency, we systematically evaluated the impact of the allosteric sodium ion by performing absolute binding free energy calculations both in the presence and absence of Na^+^ across all the A_2A_ systems (SI Table S4).

We see that calculations with the sodium ion always give better metrics when compared to calculations without the sodium ions for all the data sets of A_2A_ receptor.

Figure 5 illustrates the effect of including the allosteric sodium ion on ABFE predictions across three A_2A_ receptor data sets. For the Lenselink et al.^13^ data set (Figure 5 a,b, SI Table S4), inclusion of sodium resulted in a clear improvement in predictive performance, reducing the mean unsigned error (MUE) from 1.22 to 0.91 kcal mol^–1^ and the RMSE from 1.44 to 1.08 kcal mol^–1^. Improvements were also observed in correlation metrics, with the Pearson and Spearman coefficients increasing from 0.72 to 0.90 and from 0.61 to 0.82, respectively.

**Figure 5:**
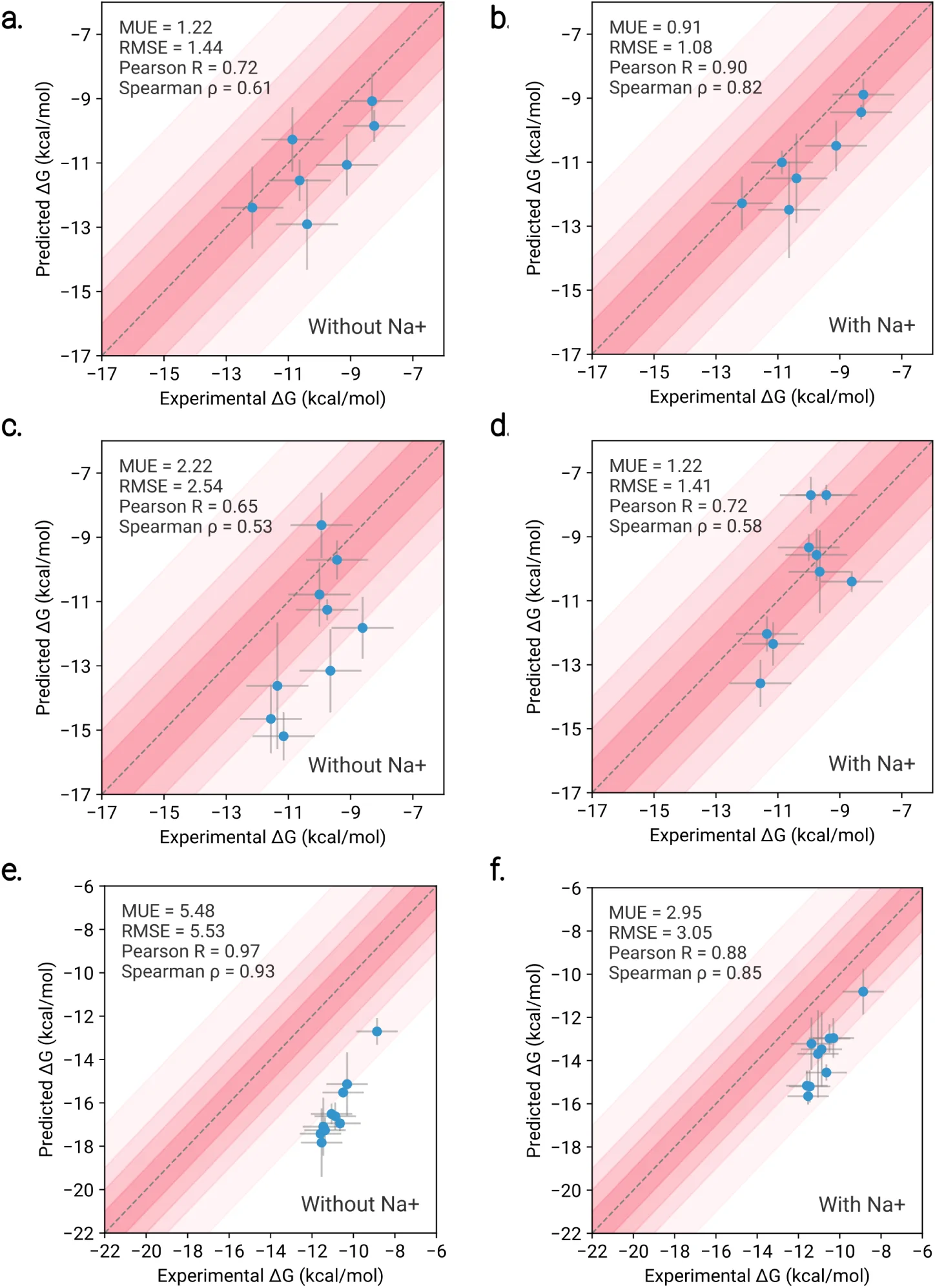
Impact of bound sodium on ABFE predictions across A2A receptor data sets. (a, b) ABFE results are shown for the Lenselink et al., (c, d) Minetti et al., and (e, f) Deflorian et al. data sets with sodium unbound (a, c, e) and sodium bound (b, d, f).

A similar trend was observed for the Minetti et al.^52^ data set (Figure 5 c,d, SI Table S4). Inclusion of sodium substantially improved accuracy, with the MUE decreasing from 2.22 to 1.22 kcal mol^–1^ and the RMSE from 2.54 to 1.41 kcal mol^–1^. Corresponding improvements in correlation metrics were also observed, indicating better ranking of compounds.

For the Deflorian et al.^2^ data set (Figure 5 e,f, SI Table S4), which spans a narrower experimental affinity range, inclusion of sodium again led to improved agreement with experiment. In this case, a pronounced reduction in the overestimation of binding affinities was observed, with the MUE decreasing from 5.48 to 2.95 kcal mol^–1^ and the RMSE from 5.53 to 3.05 kcal mol^–1^. These results suggest that explicit inclusion of sodium preferentially stabilizes the apo receptor state, thereby shifting the conformational ensemble away from an inactivated (holo-like) state toward an heterogeneous apo-like state more consistent with experimental binding measurements.

Although a reduction in correlation coefficients was observed, they remained high overall, with the Pearson correlation decreasing from 0.97 in the absence of sodium to 0.88 when sodium was included.

### Allosteric Sodium as a Source of Systematic Bias in ABFE Calculations of the D3 Receptor

Like other class A GPCRs, the dopamine D3 receptor (D3R) contains a conserved allosteric Na^+^ binding site located beneath the orthosteric pocket. This site is centered on Asp^2.50^ (Asp72 in D3R numbering) and coordinates a network of water molecules via the side-chain carboxylate of Asp^2.50^ and the hydroxyl group of Ser^3.39^ (Ser114 in D3R). Although no experimental D3R structure has been resolved with a bound sodium ion, the cavity observed in the eticlopride-bound D3R crystal structure^53^ closely superimposes with Na^+^ binding sites identified in other aminergic GPCRs, including the β_1_- and β_2_-adrenergic and A_2_A adenosine receptors. To validate the functional role of the cavity, Michino et al.^54^ explicitly modeled Na^+^ into this cavity and performed long-timescale molecular dynamics simulations of the resulting D3R–ligand complexes. In these simulations, the ion remained stably coordinated with sodium binding stabilizing a specific rotameric state of Ser^3,39^ leading to a stabilized inactive-like conformation of the binding pocket. In contrast, simulations performed in the absence of Na^+^ exhibited increased conformational heterogeneity of side chain movement.

Experimental studies have further demonstrated that sodium ions enhance the binding affinity of D3R for several antagonists and inverse agonists, including SB-277011-A, L-741,626, GR103691, and U99194.^55,56^ In contrast, the affinities of some ligands like spiperone and (+)-butaclamol appear insensitive to sodium while the affinity of other ligands like clozapine seems to be sensitive to the overall ionic strength rather than to specific cation effects. Although these compounds are structurally distinct from the SAR series reported by Kumar et al.,^56^ (SI Table S5), the documented sodium dependence of several known antagonists together with the absence of a resolved Na^+^ ion in the structures reported by Guo et al. motivated us to examine the role of sodium binding in modulating D3R ligand affinity. Therefore, we performed absolute binding free energy (ABFE) calculations using receptor models with and without a bound Na^+^ ion. The Na^+^ -bound D3R model was generated by transferring the ion from the A_2A_ receptor structure (PDB ID: 4EIY)^57^ into our D3R model after superimposing the structures.

ABFE calculations performed with Na^+^ unbound (Figure 6a) and Na^+^ bound (Figure 6b) systems show comparable performance, with RMSE values of 2.93 and 2.94 kcal/mol, respectively. The Na^+^ bound system yields marginally better absolute error (MUE of 2.22 vs 2.33 kcal/mol) but slightly worse linear agreement (Pearson R of 0.34 vs 0.41), while compound ranking remains identical (Spearman ρ = 0.43 in both cases). Overall, Na^+^ inclusion has a small effect on D3 receptor calculations, with no observed drawback, though the small sample size limits the strength of this conclusion.

**Figure 6:**
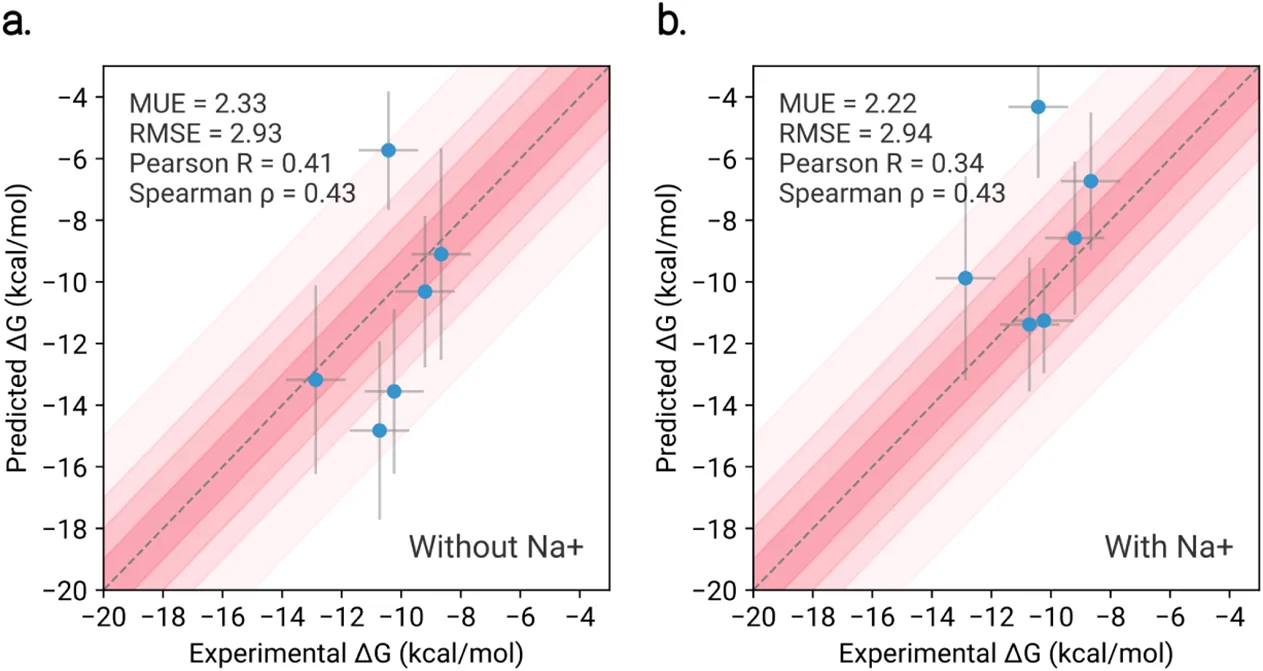
Impact of bound sodium on ABFE predictions for the D3 receptor (Kumar et al.^56^ data set). (a) ABFE results calculated without the bound sodium. (b) ABFE results calculated with the bound sodium.

These results align with the established role of Na^+^ in entropically stabilizing the apo receptor, which is expected to reduce antagonist binding affinity and mitigate the observed overestimation of ABFE values. Therefore, from these results for the A_2A_ and D3 receptors, we conclude that while including suspected bound ions is advisable, this cannot be universally generalized and should be applied with caution on a system-by-system basis (see SI Table S6).

### Incomplete Apo State Relaxation as a Source of Systematic Error in ABFE Calculations of the A2A Receptor

We observed that the performance of ABFEs was considerably poorer on the A_2A_R data set from Deflorian et al.^2^ The best-performing ABFE protocols achieved RMSE values of 1.08 and 1.41 kcal/mol on the Lenselink^13^ and Minetti^52^ A_2A_R data sets, respectively. In contrast, performance on the Deflorian et al. data set was substantially worse, with an RMSE of 3.2 kcal/mol (Figure 5f). Notably, this deviation presented as a nearly uniform overestimation across all the compounds, indicating a systematic bias. Our first instinct was to look for a resolvation problem of the kind we had identified in mGlu5, but it quickly became clear that the A2A binding pocket is open to solvent and does not suffer from the same exchange limitations (SI Figure S4). We therefore propose a different hypothesis: that following ligand decoupling, the receptor becomes kinetically trapped in a holo-like inactive conformation and cannot relax toward an apo-like ensemble within the time scales accessible to the simulation.

In order to quantify the apo-likeness of the receptor in our ABFE simulations, we examined existing literature on the structural differences between the apo and the holo-inactivated ensembles of the receptor. Multiple studies on the differences between the holo-inactivated and the apo like state of the receptor^58^ have shown that the apo A_2A_ receptor has a more heterogeneous ensemble, whereas antagonist binding stabilizes a restricted inactive substate.

At the level of the transmission network, the antagonist-bound state exhibits a broken ionic lock (R102^3.50^-E228^6.30^) stabilized in a fixed conformation, while the apo receptor fluctuates between formed and broken states. Similarly, the rotamer toggle switch (T88^3.36^/F242^6.44^/W246^6.48^) adopts ligand-stabilized rotamers in the antagonist-bound state but samples multiple conformations in the apo ensemble. The conserved NPXXY motif residue Y288^7.53^ which mediates TM7 rearrangement, also exhibits greater conformational diversity in the apo state compared to its more rigid inactive-like orientation in the presence of antagonist. These distinctions indicate that even the inactive ligand-bound state is conformationally distinct from the apo ensemble.

Given the difficulty of exhaustively sampling such a heterogeneous apo ensemble within accessible simulation time scales, we instead aimed to identify a more relaxed receptor conformation that is energetically lower than the initial holo-inactivated state. To this end, we performed three independent 1 μs simulations of the apo receptor, starting from the holo-inactivated structure 6GT3.^59^

To quantitatively distinguish between the different conformations of the receptor binding pocket, we adopted an approach inspired by Vögele et al.^23^ In their study, receptor binding pockets were featurized using pairwise Cα distances and projected onto a principal components space to differentiate active and inactive GPCR states. We implemented a similar approach (see Methods) to characterize conformational variability in A_2A_R. This analysis enabled us to identify distinct metastable states from our simulations.

Projection of the apo, holo, and ABFE trajectories onto the principal component space revealed that the ABFE simulations predominantly overlapped with the holo ensemble rather than the apo ensemble (SI Figure S6 and SI Figure S7) This pattern was consistent across all compounds in the Deflorian data set. Repeating this analysis for the remaining data sets, Lenselink et al., Minetti et al., and Kumar et al., showed reasonable overlap between the apo ensemble and the final window of the ABFE trajectory, further supporting our hypothesis (SI Figure S8). The Deflorian set, on the other hand, shows much worse overlap (SI Figure S9).

These findings support our hypothesis that the receptor remains confined to a holo-like conformational basin following ligand decoupling. Such incomplete relaxation would bias the binding free energy estimate by neglecting the energetic cost associated with receptor conformational relaxation, thereby leading to systematic overestimation of affinity.

If we can quantify the free energy of relaxation from the holo-like state to the apo-like state of the receptor $\left(\Delta G_{holo\to apo}^{receptor,REUS}\right)$ , (Figure 7a) this term can be incorporated as a correction to the FEP-derived annihilation free energy of the complex $\left(\Delta G_{annihil.}^{complex,FEP}\right)$ . Adding this correction should yield an improved estimate of the absolute binding free energy $\left(\Delta G_{annhil.}^{complex,ABFE}\right)$ (Figure 7a).

**Figure 7:**
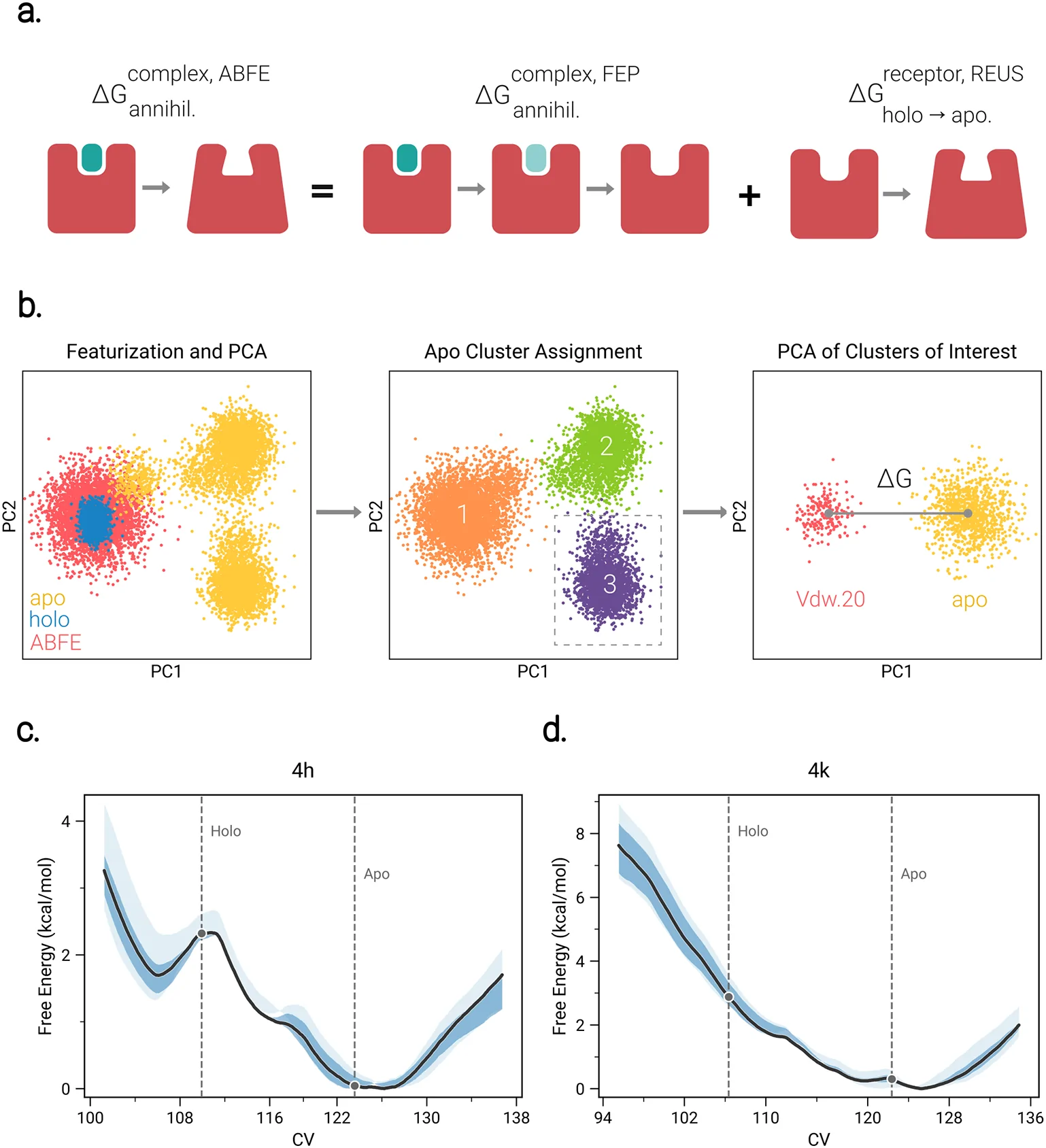
Correction of systematic overestimation in the Deflorian A2AR data set: (a) Thermodynamic scheme illustrating incorporation of receptor relaxation (Δ*G* receptor, REUS holo→apo) as an additional leg to correct the FEP-derived annihilation free energy. (b) Workflow for construction of the collective variable (CV) used in the REUS simulations. The binding pocket was first featurized using pairwise Cα distances and projected onto principal component (PC) space. Clustering in this space identified distinct metastable states, including apo-like conformations. When multiple apo clusters were present, the cluster with the highest population was selected (Figure 7b – purple box). Structures from the apo cluster and from the final leg of the ABFE simulations (vdw.20) were then combined and used to perform a refined PCA. In most cases, a single principal component captured the dominant variance between the two ensembles and was used as the 1D CV for REUS. (c) Free energy surfaces (FES) obtained from REUS simulations of compound 4h with two distinct wells for apo-like and holo-like states and (d) FES for compound 4k without a clear barrier separating the states.

To estimate the $\Delta G_{holo\to apo}^{receptor}$ , we computed the free energy difference between the receptor conformation in the final window of the ABFE simulation and the receptor conformation from the long apo simulation using replica exchange umbrella sampling. Because convergence of REUS critically depends on the choice of collective variable (CV), we systematically evaluated candidate CVs before selecting the CV described in Figure 7b. The CV was constructed by selecting structural features that most strongly differentiated the two ensembles and projecting them onto their principal components (SI Figure S10) (see Methods). This reduced representation provided a 1D CV that was used to perform an umbrella sampling simulation between the two states.

We performed REUS simulations for each compound using a collective variable derived from projecting the ABFE trajectories of the ligand together with the apo trajectories. These simulations were used to estimate the free energy difference between the receptor conformation sampled in the final stage of the ABFE calculations and an apo-like conformation. This procedure was repeated independently for all 10 compounds in the Deflorian data set.

The selected CV successfully identifies a lower-energy receptor state distinct from the conformation sampled during the final leg of the ABFE simulations. The free energy surfaces (FES) reconstructed from the REUS reveal that the vdw.20 state representing the ABFE ensemble typically lies away from the global minimum (SI Figure S11). In contrast, the apo ensemble generally occupies regions closer to the energetic minimum. However, this is not universal, and there are some compounds (4j, 4n) where the apo cluster lies farther away from the energetic minima (Figure S11d,h). Importantly, regardless of the relative position of the apo cluster, the CV consistently identifies a lower-energy basin distinct from the ABFE state.

The FES reconstructed from the REUS simulations generally exhibit one of two qualitative profiles (SI Figure S12). In the less common case, the surface displays two distinct wells separated by an energetic barrier, corresponding to a holo-like basin (sampled during the ABFE simulations) and a lower-energy apo-like basin (e.g., Figure 7c). More frequently, the FES exhibits a single dominant minimum without a clearly defined barrier, with the ABFE ensemble positioned away from this minimum (e.g., Figure 7d).

In both scenarios, the ABFE ensemble typically occupies a region higher in free energy relative to the global minimum. We therefore define the receptor relaxation free energy correction as the difference between the free energy at the centroid of the ABFE ensemble in CV space and the global minimum of the FES (set to zero). This quantity is then used to adjust the corresponding ABFE binding free energies of the compounds.

### REUS-Based Relaxation Correction Reduces Systematic Overestimation in A2A ABFE Calculations

Incorporating the apo relaxation correction terms into the binding affinity predictions from FEP considerably improved the agreement of affinity estimates to the experiment (Figure 8), bringing the RMSE down from 3.2 to 1.28 kcal/mol. Although the corrected affinities are still overestimated, with a mean signed error of 0.8 kcal/mol, the systematic error is significantly reduced. However, while the absolute affinity estimates have improved, the performance in ranking of the compounds has deteriorated compared to the uncorrected estimates as exemplified by the Spearman coefficient going from 0.83 to 0.59. This likely reflects the propagation of uncertainties associated with performing independent REUS calculations for each compound, which introduce compound-specific variability and affect the relative ordering.

**Figure 8:**
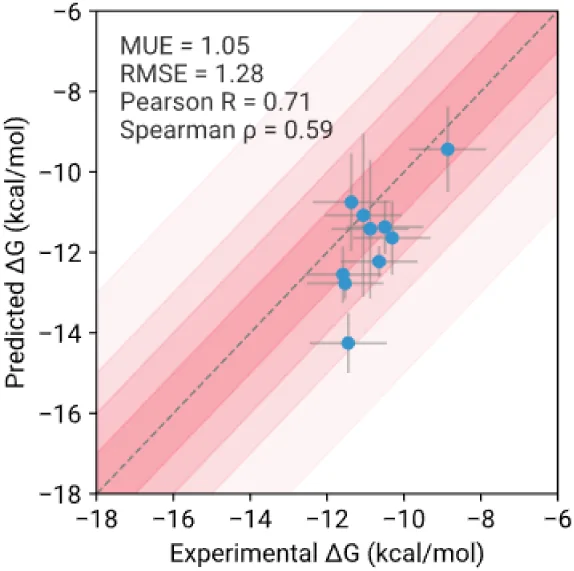
Improvement in ABFE binding affinity predictions following compound-specific receptor relaxation correction.

Given that the initial overestimation in the Deflorian A_2A_R data set was largely uniform across compounds, we explored the use of a single global correction term derived from the data collated from all the holo and ABFE trajectories applied to all ligands. In principle, such a uniform correction should mitigate systematic overestimation without introducing additional compound-wise errors.

To test this, we applied the correction using the collated data set, and the results are shown in Figure 9. The free energy surface (Figure 9A) was well converged, with the apo state located closer to the free energy minimum, while the holo state remained farther from the minimum, consistent with previous observations. The resulting free energy correction was approximately 3 kcal mol^–1^, which closely matches the systematic error observed in the initial ABFE estimates. Applying this correction reduced the RMSE of the ABFE predictions to 0.78 kcal mol^–1^ (Figure 9B).

**Figure 9:**
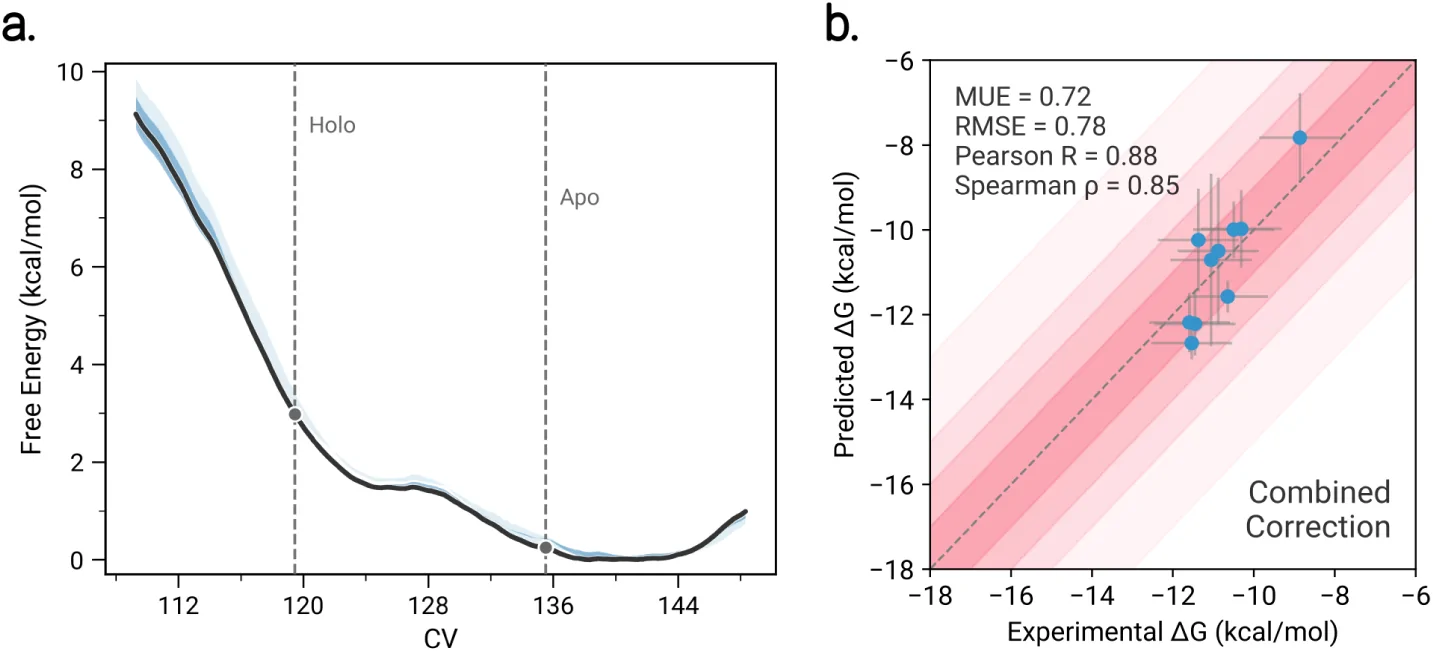
Conformational free energy contributions to ABFE calculations. (a) Free energy surfaces (FES) obtained from replica-exchange umbrella sampling (REUS) simulations of the combined data set, showing the transition from the Holo like structure from the non interacting ABFE window identified from long apo simulations. (b) Improvement in ABFE binding affinity predictions following a single receptor relaxation correction for the combined data set.

In this case, the correction from the collated data set proved effective because the overestimation in the data set was largely compound independent. However, if the overestimation were compound dependent, the effectiveness of this method would likely be limited (see SI Table S7).

## Discussion

Our results indicate that, although ABFE calculations perform well for certain systems out of the box, they remain highly sensitive to system setup and the extent of sampling available.^5,60^ While we have focused here on the role of treatment of ions, multiple other aspects of system preparation may also influence ABFE performance, particularly for systems that have not been previously studied using free energy perturbation (FEP) methods. Previous studies have highlighted additional sources of sensitivity, including the placement of interfacial water molecules, the selection of protein side-chain rotamers, and the initial ligand binding pose, all of which can substantially impact calculated free energies.^61^ In this study, the use of systems that had already been explored with relative binding free energy (RBFE) approaches likely mitigated some of the challenges associated with initial setup, but even in these cases we identified significant issues.

Although one of the key advantages of ABFE is not being limited by a congeneric ligand series, in this study we were focused on a direct comparison to previous RBFE results to better facilitate a direct interpretation of potential issues. In this study we have several A2A receptor series from different studies. Pooling the analysis across all three data, although possible (SI Figure S13), can inflate apparent model accuracy (as it does here) even when individual data sets perform less well (see discussion on this by Mey et al.^48^). Thus, we have retained the separate analysis throughout this work.

In contrast to system setup, which primarily affects the relative ranking of compounds, insufficient sampling of the apo state typically impacts the magnitude of predicted affinities, often leading to systematic overestimation. These sampling deficiencies can arise in several ways. For example, inadequate resolvation of the binding site, as observed for mGlu5, or incomplete sampling of apo conformations, such as in the A_2A_ receptor, can lead to biased free energy estimates. More generally, slow conformational transitions, including side-chain rotamer rearrangements, may limit sampling of the apo ensemble, overstabilize the bound state, and contribute to overestimation of binding affinities.^6,10^

In principle, sampling limitations could be mitigated using replica exchange-based enhanced sampling approaches such as REST2. However, their application is not always feasible, as these methods require parallel sampling across multiple ABFE windows and substantial computational resources, including access to large CPU clusters or GPU systems with fast internode communication. As a result, in more constrained computational settings, inadequate sampling of the apo state may be further exacerbated.

Challenges with sampling highlight the value of carefully assessing the apo state prior to undertaking ABFE calculations. As a rule of thumb, initial simulations of the apo receptor can provide insight into potential sources of error and guide protocol design. Key aspects to examine include binding pocket solvation, side-chain rotamer changes, and differences between apo and bound conformational ensembles. Dimensionality reduction techniques were useful for identifying distinct conformational states in the apo ensemble. While methods such as UMAP and t-SNE captured more detailed clustering, PCA was ultimately preferred due to its interpretability and suitability as a collective variable in REUS simulations. Once sources of error are identified, targeted protocol adjustments can be applied, such as using REUS to estimate apo state relaxation or GCMC-like approaches to improve binding pocket resolvation.

The two correction strategies employed here, GCMC-like sampling to improve resolvation of initial structures for alchemical windows, and REUS to estimate the free energy associated with relaxation to the apo state, performed reasonably well for the Christopher et al. mGlu5 and Deflorian et al. A_2A_ data sets, respectively. In the case of mGlu5, RMSE was reduced from 8.31 to 4.89 kcal mol^–1^, although a residual bias remained, the origin of which is unclear, but may reflect the influence of the extracellular domain on the underlying energy landscape. Further work beyond the scope of this paper would be required to investigate that in detail. In the case of Deflorian et al., RMSE was reduced from 3.05 to 0.78 kcal mol^–1^. If resources are not limiting, the GCMC protocol could be applied regardless to any prospective campaign with the expectation that it would not worsen any outcomes. Indeed, as expected applying this to the Lenselink et al. data set shows no significant change in the results (SI Figure S5). It is also worth pointing out that incorporation of GCMC in intermediate alchemical states may also yield further improvements, but such implementation is not trivial and thus may be an aspect to investigate in future studies. It is worth noting also that resolvation does not appear to materially affect phase-space overlap along the alchemical path (SI Table S8).

Both approaches have notable limitations. Our implementation of the GCMC-like resolvation protocol introduces additional workflow complexity, requiring conversion and reprocessing of the system in OpenMM (in the current implementation), followed by multiple iterations to obtain a suitably solvated structure. Similarly, the effectiveness of REUS in capturing conformational relaxation depends strongly on the quality of the apo ensemble. In this work, the apo simulations reproduced relevant conformational states, enabling meaningful free energy estimates; however, this is not always guaranteed. Apo ensembles are often heterogeneous, and simulations may fail to sample the relevant low-energy states. When multiple apo states are identified, they must be compared and evaluated prior to selecting states for REUS-based relaxation, further complicating the analysis. In our case, multiple metastable apo states were identified and REUS was performed between them to determine the lowest-energy state; however, this approach is sensitive to factors such as hysteresis, the choice of collective variables, and the convergence of the simulations. Future work could explore the use of homology modeling or machine learning-based approaches, such as BioEmu^62^ and AlphaFlow^63^ to generate representative apo conformations.

We also examined whether the chemical nature of the ligands could predict the sampling challenges encountered. The A2A data sets (Deflorian et al., Minetti et al., and Lenselink et al.) comprise rigid, aromatic amino-azaarene/diaryl compounds with predominantly hydrophobic and lipophilic substituents. The Deflorian data set carries halogen substituents (F, Cl, CF_3_), while the Lenselink and Minetti data sets share a heteroatom-rich, fused aza-aromatic core, with lipophilicity rising sharply through large alkyl, phenethyl, and cyclohexylpropyl tails. However, ligand hydrophobicity did not correspond to resolvation issues, consistent with a solvent-exposed binding pocket (SI Figure S4). The Christopher et al. mGlu5 data set, by contrast, consists of neutral, fairly planar aryl-azaheteroaryl ligands bearing CN, F, and Cl substituents. These ligands have simple degrees of freedom and are straightforward to sample, reflected in the generally low inter-repeat errors between FEP runs.

The Kumar et al. data set proved the most challenging of the five, comprising monocationic aryl-piperazines that are intrinsically hydrophobic in their aromatic and alkyl character. Their net charge introduces additional complications for the alchemical calculations, which we address by including an alchemical ion.^48,64^ However, the ligand can be slow to equilibrate across perturbation windows, increasing the sampling required for convergence. This manifests as substantial differences between individual ABFE repeats, up to 7 kcal/mol. Although the combination of hydrophobicity and slow charge equilibration might suggest solvation issues, this is only partly borne out: charge and hydrophobic character together complicate resolvation, producing wide variance across repeats (SI Figure S4), though the pocket remains well solvated on average. Because the pocket is solvent-facing, we did not pursue additional GCMC sampling for this system.

It is worth noting that this study focused on antagonists and extending these approaches to agonists, which often induce larger conformational transitions toward active states, is likely to be more challenging. Moreover, the performance of the REUS-based correction method depends on the coupling between the proposed CV and the conformational change it induces.

It is also worth highlighting that alternative strategies to improve apo state sampling, such as extending ABFE simulation time scales (e.g., to ∼100 ns or longer), may or may not be beneficial and will likely be context dependent. Previous studies have shown that increased simulation length alone does not guarantee complete exploration of the conformational landscape and while longer simulations can improve sampling, they are not a substitute for targeted approaches that address specific sources of under-sampling.^6^

Across both the A2A and D3 data sets, including Na^+^ consistently reduced the overestimation of antagonist binding affinity seen in Na^+^-free calculations. This would imply that Na^+^ preferentially stabilizes the neutral apo state over the antagonist bound inactivated-like state.

This sits in apparent tension with the established role of Na^+^ as a negative allosteric modulator (NAM), which favors the inactive state and should increase antagonist affinity. We do not think the two are contradictory. The NAM effect describes how Na^+^ shifts the true conformational equilibrium of the receptor, whereas our ABFE protocol treats Na^+^ occupancy as a constant throughout the thermodynamic cycle, rather than as an equilibrium coupled to receptor conformation. This coupling between ion occupancy, ligand binding, and conformational transitions could be stronger still in the case of agonists. A rigorous treatment would therefore require explicitly accounting for the free energy of sodium binding or release using enhanced sampling methods, rather than assuming identical sodium occupancy between states. Restrictions on available compute and the complexity of probing such a system make proper characterization of ion behavior difficult. Currently, the question of whether to include allosteric ions in ABFE calculations remains one that has to be answered on a case-by-case basis, unless there is clear experimental evidence favoring the bound or unbound state.

A comparison of ABFE and RBFE ranking performance across all data sets is summarized in Table 1. Overall, ABFE methods demonstrate utility, with Pearson correlations comparable to RBFE; however, performance across other absolute metrics remains less consistent. These results suggest that, while ABFE can be a powerful approach, its reliability depends critically on careful system setup, sufficient sampling, and appropriate methodological corrections. All of these components are typically canceled out with RBFE approaches. However, quite often, in the absence of experimental ground-truths, ABFE calculations are used to anchor cycle closures of RBFE studies. Thus, particular care should be exercised in those calculations to ensure predictive value is maximized.

**Table 1:** Performance Metrics for the Best ABFE Estimates across Five Published Data Sets after Applying the Data Set-Specific Improvements Described in This Work^a^

Data set	N	MUE (CI)	RMSE (CI)	R^2^ (CI)	Pearson *r* (CI)	Spearman *ρ* (CI)	Kendall *τ* (CI)
Christopher et al. mGlu5	12	4.45 (3.24–5.51)	4.89 (3.96–5.67)	–4.03 (−11.29––2.30)	0.42 (−0.04–0.94)	0.57 (−0.04–0.94)	0.45 (0.00–0.86)
Deflorian et al. A2AR	10	0.72 (0.53–0.91)	0.78 (0.58–0.95)	0.00 (−4.83–0.46)	0.88 (0.37–0.98)	0.85 (0.39–1.00)	0.69 (0.23–1.00)
Kumar et al. D3R^b^	6	2.22 (0.92–3.85)	2.94 (1.03–4.51)	–3.84 (−55.01––0.24)	0.34 (−0.77–0.99)	0.43 (−0.74–1.00)	0.33 (−0.50–1.00)
Lenselink et al. A2AR^b^	7	0.91 (0.48–1.33)	1.08 (0.65–1.44)	0.35 (−2.24–0.81)	0.90 (0.59–0.99)	0.82 (0.06–1.00)	0.71 (0.06–1.00)
Minetti et al. A2AR^b^	9	1.22 (0.75–1.68)	1.41 (0.93–1.78)	–1.29 (−6.99––0.23)	0.72 (0.12–0.97)	0.58 (−0.32–0.95)	0.39 (−0.38–0.88)

a Values are estimates with bootstrap confidence intervals (10,000 resamples).

b Small sample size (*N* < 10).

## Conclusions

Part of what makes ABFE calculations, and alchemical free energy methods more broadly, so appealing is that the alchemical coordinate naturally drives the system between bound and unbound states, acting as an effective collective variable without requiring explicit knowledge of the unbinding pathway. This sets it apart from path-based approaches such as funnel metadynamics. But this strength comes with an inherent limitation: the alchemical variable couples poorly to degrees of freedom that relax slowly relative to the time scales accessible in a typical ABFE simulation. Solvation of the binding pocket, conformational relaxation of the receptor, and ion binding and unbinding can all fall into this category, and when they do, the system does not have sufficient time to equilibrate as the alchemical variable is perturbed. In this work, we identified all three as consequential sources of error in ABFE calculations in GPCR systems.

We have shown that careful treatment of solvation, especially in the case where the pocket is deep and water exchange is likely to be difficult, can significantly improve ABFE estimates even in the presence of other errors. Accounting for conformational change as a separate contribution is likely to be a generally useful approach going forward and we therefore recommend incorporating this step in future protocols, even in cases where the changes between apo and holo appear small. The importance of allosteric ions (or other cofactors) and the question of including them in the system, are difficult to resolve at this point with calculations showing mixed results. Nevertheless, the error associated with the ABFE calculations remains considerable underscoring the need for further investigation and methodological improvement. Regardless, it remains the case that ABFE calculations are sensitive to many different factors that vary in terms of their influence on a case-by-case basis. Use of automated and pipelined ABFE calculations should proceed with extreme caution.

## Supplementary Material



## Data Availability

The input structures and dH/dλ files for all five GPCR data sets and the data obtained from the REUS simulations are publicly available on Zenodo at 10.5281/zenodo.19898263. The code for the biased GCMC-like water transport scheme is available at https://github.com/Nithishwer/grand/tree/alchemical_ligand, and the code for performing REUS-based conformational free energy calculations is available at https://github.com/Nithishwer/FEPA/tree/main.

## #

Nuvisan, Müllerstraße 178, 13353 Berlin, Germany

## &

Yale University, New Haven, Connecticut 06520, USA.

## Abbreviations

A_2A_R: Adenosine A_2A_ Receptor
mGlu5: metabotropic glutamate receptor
PCA: principal component analysis
GCMC: Grand Canonical Monte Carlo
MUE: mean unsigned error
RMSE: root-mean-squared error
ABFE: absolute binding free energy
FEP: free energy perturbation
