# Supplementary material for: Improving ABFE Calculations for GPCRs by Addressing Errors in Apo-State Representation
Source: J Chem Theory Comput. 2026 Sep 28;22(19):10428–44. doi: 10.1021/acs.jctc.6c01161 (PMC13671163; doi:10.1021/acs.jctc.6c01161)
Supplement: Supplementary file 1 [file ct6c01161_si_001.pdf]

Supporting Information

Improving ABFE Calculations for GPCRs by Addressing Errors in Apo-State Representation

*Nithishwer Mouroug Anand,[1] Antonia-Florina Panescu,[1] Ronald Knegtel,[2] Ewa Chudyk,[2,3] Fabio Zuccotto[2] and Philip C. Biggin[1]**

Dataset	RMSE	MUE	R^2	Kendal τ	Ligands / edges	Notes	DOI
Deflorian A2A	1.58 (p); 1.22 (e)	1.21 (p); 1.00 (e)	0.5	0.49	17 / 28	Study included extra ligands from the Deflorian et al. paper.	https://doi.org/10.1038/s42004-023-01019-9
Lenselink A2A	0.97	0.9	0.58	0.62	7 / NA	Recalculation from SI Table S4 starting from PDB 3PWH, but not as reported.	https://doi.org/10.1021/acsomega.6b00086
Lenselink A2A	1.75	1.47	0.19	0.33	7 / NA	Recalculation from SI Table S4 starting from PDB 4EIY, but not as reported.	https://doi.org/10.1021/acsomega.6b00086
Minetti A2A	-	0.68	0.61	-	9 / 18	From Table 1.	https://doi.org/10.1021/acsomega.6b00086
Minetti A2A	0.55	0.45	0.82	-	9 / 16	From Table S3.	https://doi.org/10.1021/acs.jcim.4c00764

Table S1. FEP performance for A_2A receptor ligand datasets from existing studies other than Guo et al. Errors are in kcal mol^{-1}; "–" indicates unavailable values. Direct comparisons should be interpreted cautiously because ligand sets, receptor structures, FEP protocols, and metric definitions differ. (p) indicates pairwise, (e) indicates edgewise.

Ligand	Canonical isomeric SMILES	Structure	Predicted ΔG, noHREX (kcal mol^{-1})	Predicted ΔG, HREX (kcal mol^{-1})	Predicted ΔG, apo (kcal mol^{-1})	Predicted ΔG, EHREX (kcal mol^{-1})	Experimental ΔG (kcal mol^{-1})
1	CCCCc1nc(N)c2nc(-n3nccn3)n(C)c2n1	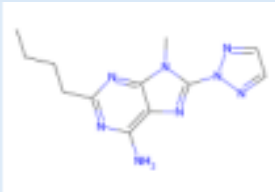	-8.05 ± 2.01	-10.27 ± 1.00	-6.45 ± 2.45	-7.76 ± 0.75	-10.87
11	Cn1c(-n2nccn2)nc2c(N)nc(C(=O)Nc3ccccc3)nc21	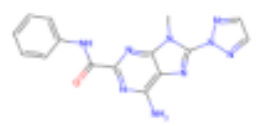	-11.18 ± 1.58	-12.91 ± 1.42	-9.15 ± 1.56	-10.34 ± 1.34	-10.4
13	CN(C)c1nc(N)c2nc(-n3nccn3)n(C)c2n1	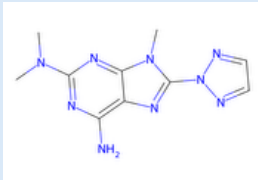	-5.51 ± 1.95	-9.07 ± 0.86	-5.17 ± 2.38	-6.43 ± 0.91	-8.31
17	Cn1c(-n2nccn2)nc2c(N)nc(NC(=O)c3ccccc3)nc21	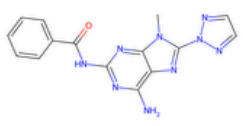	-9.63 ± 3.17	-11.06 ± 0.95	-6.04 ± 2.81	-8.45 ± 0.61	-9.12
2	CC(C)c1nc(N)c2nc(-n3nccn3)n(C)c2n1	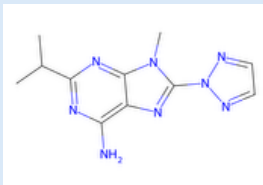	-7.67 ± 2.03	-9.85 ± 0.50	-6.38 ± 0.51	-7.8 ± 0.44	-8.24
3	Cn1c(-n2nccn2)nc2c(N)nc(CCc3ccccc3)nc21	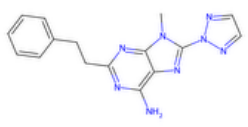	-10.17 ± 1.95	-11.54 ±0.64	-7.88 ± 1.73	-8.41 ± 0.78	-10.64
8	Cn1c(-n2nccn2)nc2c(N)nc(CCC3CCCCC3)nc21	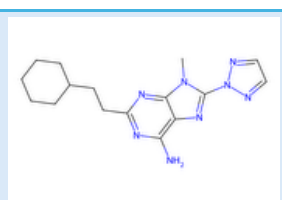	-11.5 ± 2.9	-12.39 ± 1.28	-7.15 ± 3.10	-8.74 ± 0.76	-12.16

Table S2. Ligand-specific absolute binding free-energy results for the Lenselink A2A receptor dataset. Canonical isomeric SMILES and 2D chemical structures are provided for each ligand. Predicted binding free energies (ΔGpred) and their standard deviations across replicate calculations are reported for conventional sampling without HREX (noHREX), Hamiltonian replica exchange (HREX), apo-state simulations (apo), and energy-HREX (EHREX); experimental binding free energies (Δ Gexp) are shown for comparison. All free energies are in kcal mol-1.

Ligand	Canonical isomeric SMILES	Structure	Predicted ΔG, unsolvated (kcal mol^{-1})	Predicted ΔG, solvated (kcal mol^{-1})	Experimental ΔG (kcal mol^{-1})
6	N#Cc1cc(F)cc(-c2cc(-n3cccn3)ncn2)c1	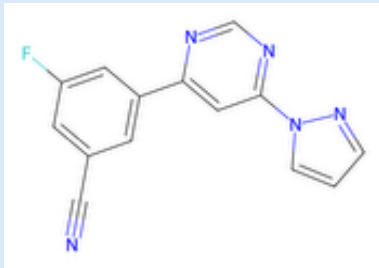	-19.19 ± 1.47	-15.49 ± 1.35	-9.82
7	N#Cc1ccc(F)c(-c2cc(-n3cccn3)ncn2)c1	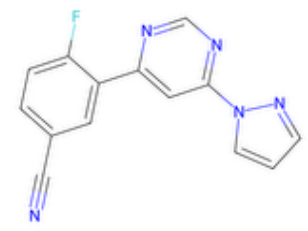	-17.69 ± 2.74	-14.4 ± 3.02	-9
8	N#Cc1cccc(-c2cc(-n3cccn3)ncn2)c1	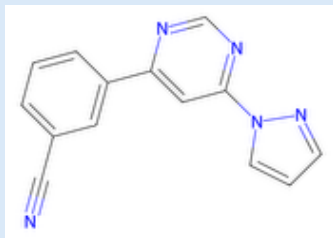	-17.57 ± 2.18	-13.38 ± 1.38	-8.32
9	Fc1cccc(-c2cc(-n3cccn3)ncn2)c1	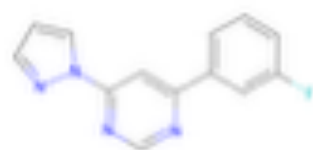	-16.41 ± 5.04	-11.74 ± 2.47	-6.95
10	COc1cc(F)cc(-c2cc(-n3cccn3)ncn2)c1	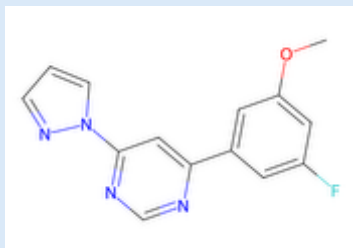	-15.78 ± 3.03	-12.92 ± 1.20	-5.73
11	NC(=O)c1cc(F)cc(-c2cc(-n3cccn3)ncn2)c1	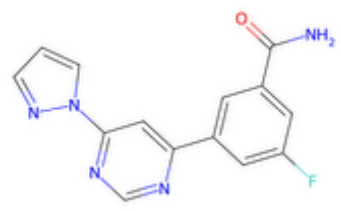	-15.49 ± 2.29	-12.25 ± 1.10	-5.73
12	Cc1cc(C#N)cc(-c2cc(-n3cccn3)ncn2)c1	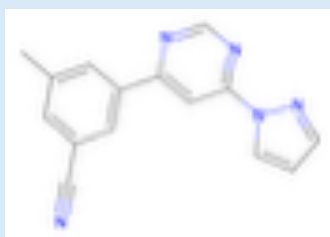	-18.41 ± 0.94	-15.59 ± 0.89	-11.45
13	N#Cc1cc(Cl)cc(-c2cc(-n3cccn3)ncn2)c1	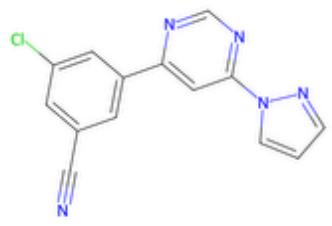	-20.57 ± 2.64	-16.79 ± 1.49	-11.45
14	N#Cc1cc(Cl)c(F)c(-c2cc(-n3cccn3)ncn2)c1	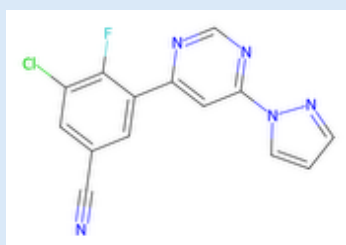	-20.52 ± 2.17	-15.64 ± 0.93	-12.68
15	Cc1cc(C#N)cc(-c2cc(-n3cccn3)ccn2)c1	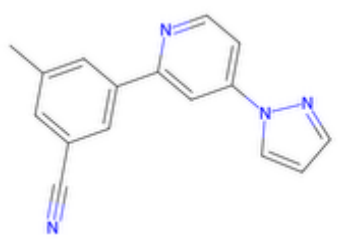	-13.91 ± 1.79	-9.27 ± 1.31	-10.36
16	N#Cc1cc(Cl)c(F)c(-c2ccnc(-n3cccn3)c2)c1	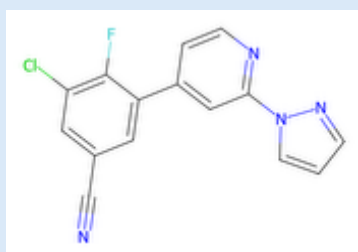	-19.67 ± 0.83	-15.68 ± 0.69	-10.5
17	N#Cc1cc(Cl)cc(-c2cc(-n3cc(F)cn3)ncn2)c1	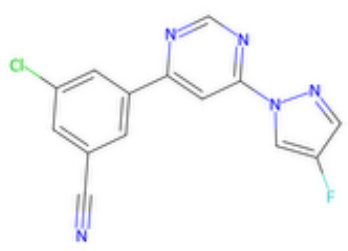	-12.34 ± 1.42	-10.41 ± 1.03	-10.5

Table S3. Ligand-specific absolute binding free-energy results for the Christopher et al. dataset. For each ligand, the table reports the ligand identifier, canonical isomeric SMILES, 2D chemical structure, predicted binding free energy from the unsolvated and solvated HREX ABFE protocols, the corresponding standard deviation across replicate TI calculations, and the experimental binding free energy. All free energies are reported in kcal mol−1.

Dataset	Ligand	Canonical isomeric SMILES	Structure	Predicted ΔG, with Na+ (kcal mol−1)	Predicted ΔG, without Na+ (kcal mol−1)	Experimental ΔG (kcal mol−1)
**Lenselink et al.**	**1**	CCCCc1nc(N)c2nc(-n3nccn3)n(C)c2n1	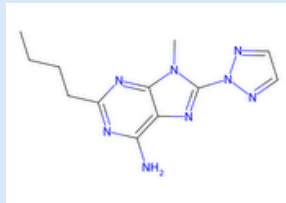	-11.01 ± 0.37	-10.27 ± 1.00	-10.87
**Lenselink et al.**	**11**	Cn1c(-n2nccn2)nc2c(N)nc(C(=O)Nc3ccccc3)nc21	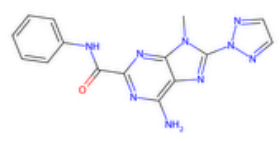	-11.5 ± 1.4	-12.91 ± 1.42	-10.4
**Lenselink et al.**	**13**	CN(C)c1nc(N)c2nc(-n3nccn3)n(C)c2n1	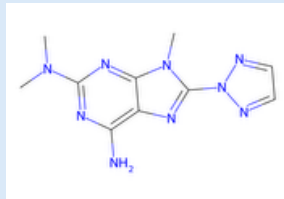	-9.44 ± 0.23	-9.07 ± 0.86	-8.31
**Lenselink et al.**	**17**	Cn1c(-n2nccn2)nc2c(N)nc(NC(=O)c3ccccc3)nc21	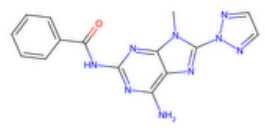	-10.49 ± 0.79	-11.06 ± 0.95	-9.12
**Lenselink et al.**	**2**	CC(C)c1nc(N)c2nc(-n3nccn3)n(C)c2n1	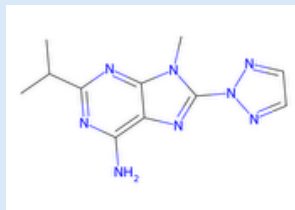	-8.89 ± 0.50	-9.85 ± 0.50	-8.24
**Lenselink et al.**	**3**	Cn1c(-n2nccn2)nc2c(N)nc(CCc3ccccc3)nc21	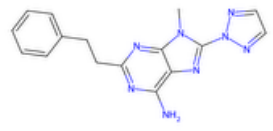	-12.49 ± 1.51	-11.54 ± 0.64	-10.64
**Lenselink et al.**	**8**	Cn1c(-n2nccn2)nc2c(N)nc(CCC3CCCCC3)nc21	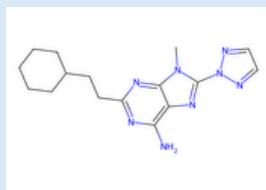	-12.28 ± 0.83	-12.39 ± 1.28	-12.16
**Minetti et al.**	**11**	Cn1c(-n2nccn2)nc2c(N)ncnc21	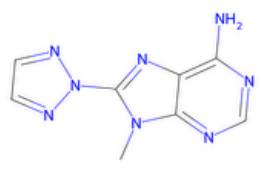	-9.34 ± 0.42	-10.78 ± 1.00	-10
**Minetti et al.**	**25a**	Cc1nc(N)c2nc(-n3nccn3)n(C)c2n1	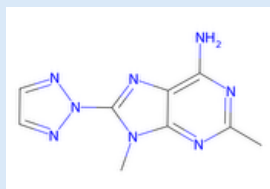	-9.57 ± 0.81	-11.25 ± 0.33	-9.75
**Minetti et al.**	**25b**	CCCCc1nc(N)c2nc(-n3nccn3)n(C)c2n1	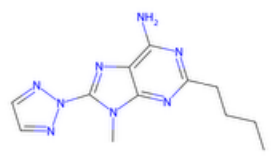	-12.35 ± 0.68	-15.19 ± 0.75	-11.15
**Minetti et al.**	**25c**	CCCCCc1nc(N)c2nc(-n3nccn3)n(C)c2n1	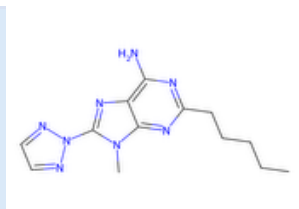	-13.58 ± 0.73	-14.65 ± 1.07	-11.56
**Minetti et al.**	**25d**	Cn1c(-n2nccn2)nc2c(N)nc(CCc3ccccc3)nc21	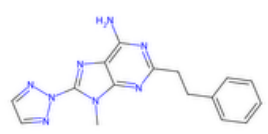	-12.04 ± 0.55	-13.62 ± 1.97	-11.35
**Minetti et al.**	**25e**	CC(C)c1nc(N)c2nc(-n3nccn3)n(C)c2n1	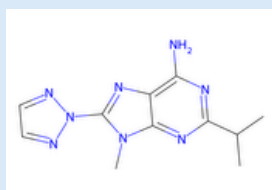	-10.4 ± 0.33	-11.82 ± 0.96	-8.61
**Minetti et al.**	**25f**	CCCc1nc(N)c2nc(-n3nccn3)n(C)c2n1	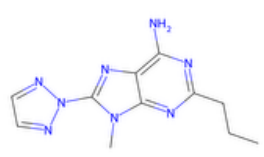	-10.09 ± 1.29	-13.15 ± 1.30	-9.65
**Minetti et al.**	**32**	Cn1c(-n2nccn2)nc2c(N)nccc21	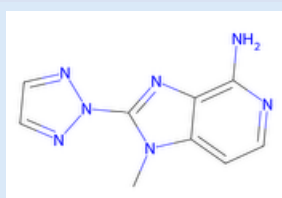	-7.7 ± 0.57	-8.62 ± 1.01	-9.94
**Minetti et al.**	**41**	Cc1cc2c(nc(-n3nccn3)n2C)c(N)n1	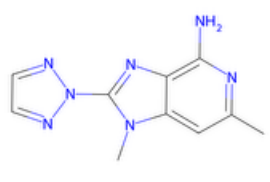	-7.7 ± 0.32	-9.7 ± 0.61	-9.44
**Deflorian et al.**	**4g**	Cc1cc(-c2nnc(N)nc2-c2ccccc2)cc(C)n1	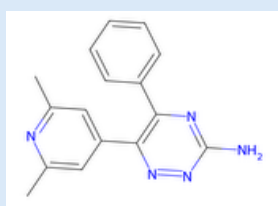	-13.69 ± 2.03	-16.51 ± 0.47	-11.06

**Deflorian et al.**	**4h**	Cc1cc(-c2nnc(N)nc2-c2ccc(F)cc2)cc(C)n1	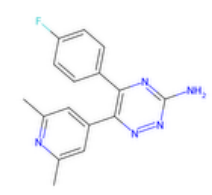	-14.55 ± 0.38	-16.94 ± 0.36	-10.65
**Deflorian et al.**	**4i**	Cc1cc(-c2nnc(N)nc2-c2cc(F)cc(F)c2)cc(C)n1	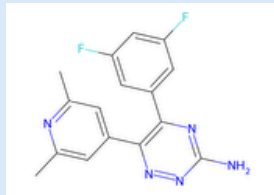	-12.96 ± 0.92	-15.14 ± 1.46	-10.31
**Deflorian et al.**	**4j**	Cc1cc(-c2nnc(N)nc2-c2cccc(F)c2)cc(C)n1	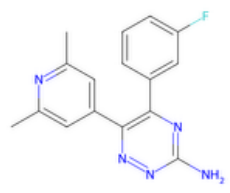	-13.48 ± 1.73	-16.61 ± 0.63	-10.88
**Deflorian et al.**	**4k**	Cc1cc(-c2nnc(N)nc2-c2ccccc2)cc(C(F)(F)F)n1	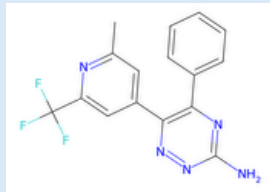	-15.65 ± 0.39	-17.83 ± 1.58	-11.53
**Deflorian et al.**	**4l**	Cc1cc(-c2nnc(N)nc2-c2ccc(F)cc2)cc(C(F)(F)F)n1	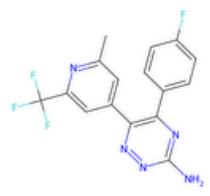	-13.22 ± 1.21	-17.26 ± 0.60	-11.37
**Deflorian et al.**	**4m**	Nc1nnc(-c2ccnc(Cl)c2)c(-c2ccccc2)n1	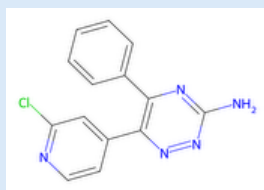	-12.97 ± 0.66	-15.52 ± 0.23	-10.5
**Deflorian et al.**	**4n**	Nc1nnc(-c2cc(Cl)nc(Cl)c2)c(-c2ccccc2)n1	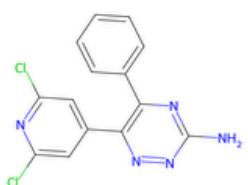	-15.16 ± 0.70	-17.42 ± 0.50	-11.59
**Deflorian et al.**	**4q**	Nc1nnc(-c2ccncc2)c(-c2ccccc2)n1	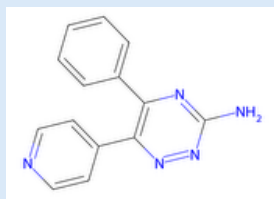	-10.81 ± 1.06	-12.71 ± 0.62	-8.86
**Deflorian et al.**	**4r**	Nc1nnc(-c2cc(Cl)nc(C(F)(F)F)c2)c(-c2ccc(F)cc2)n1	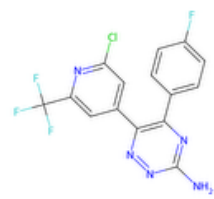	-15.2 ± 0.74	-17.1 ± 1.34	-11.45

Table S4. Ligand-specific absolute binding free energy predictions for the Lenselink et al., Minetti et al., and Deflorian et al. datasets calculated with and without Na+. Canonical isomeric SMILES, ligand structures, predicted binding free energies, (± SD), and experimental binding free energies are reported in kcal mol−1.

Ligand	Canonical isomeric SMILES	Structure	Predicted ΔG, with Na^+ (kcal mol^{-1})	Predicted ΔG, without Na^+ (kcal mol^{-1})	Experimental ΔG (kcal mol^{-1})
**11a**	CCc1cc(Cl)c(OC)c(N2CC[NH2+]CC2)c1	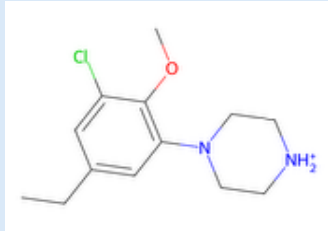	-6.73 ± 2.24	-9.1 ± 3.43	-8.66
**11b**	CCc1cccc(N2CC[NH2+]CC2)c1Cl	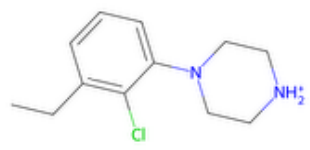	-11.26 ± 1.71	-13.55 ± 2.68	-10.24
**12a**	CCc1cc(Cl)c(OC)c(N2CC[NH+](C)CC2)c1	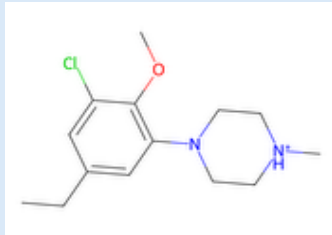	-8.58 ± 2.48	-10.31 ± 2.46	-9.2
**12b**	CCc1cccc(N2CC[NH+](C)CC2)c1Cl	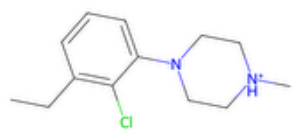	-11.38 ± 2.17	-14.82 ± 2.90	-10.73
**13a**	CCCC[NH+]1CCN(c2cc(CC)cc(Cl)c2OC)CC1	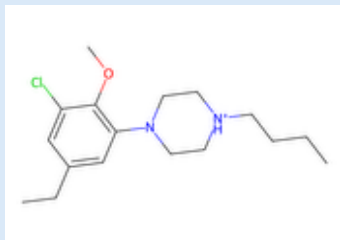	-4.32 ± 2.30	-5.73 ± 1.92	-10.43
**13b**	CCCC[NH+]1CCN(c2cccc(CC)c2Cl)CC1	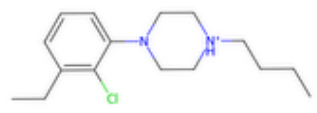	-9.88 ± 3.31	-13.18 ± 3.06	-12.87

Table S5. Ligand-specific absolute binding free energy predictions for the Kumar et al. dopamine D3 receptor dataset calculated with and without Na+. Canonical isomeric SMILES, ligand structures, predicted binding free energies, standard deviations (SD), and experimental binding free energies are reported in kcal mol^{-1}.

Receptor	Apo, ligand-free	Inactive state	Active state
**mGlu5**[1]	**No specific bound Na^+ supported**	**No specific bound Na^+ supported**	**No specific bound Na^+ supported**
**D3**[2]	**Likely bound when the apo receptor occupies an inactive-like state**; occupancy will be dynamic	**Likely bound** in the conserved class A sodium pocket	**Probably unbound** from the canonical pocket
**A2A**[3]	**Dynamically bound and shifts apo receptor toward inactive conformations**	**Bound—direct crystallographic evidence**	**Not stably bound** in the canonical pocket

Table S6. State-dependent sodium-ion occupancy in mGlu5, D3, and A2A receptors. Summary of the expected presence and stability of Na^+ in the canonical sodium-binding pocket across apo, inactive, and active receptor states, based on available structural and mechanistic evidence.

Ligand	Canonical isomeric SMILES	Structure	Predicted ΔG, global correction (kcal mol^{-1})	Predicted ΔG, compound-wise correction (kcal mol^{-1})	Experimental ΔG (kcal mol^{-1})
**4g**	Cc1cc(-c2nnc(N)nc2-c2ccccc2)cc(C)n1	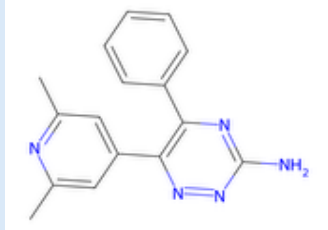	-10.71 ± 2.03	-11.08 ± 2.03	-11.06
**4h**	Cc1cc(-c2nnc(N)nc2-c2ccc(F)cc2)cc(C)n1	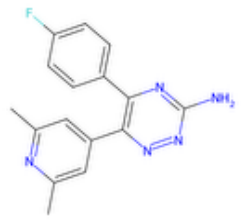	-11.57 ± 0.38	-12.23 ± 0.38	-10.65
**4i**	Cc1cc(-c2nnc(N)nc2-c2cc(F)cc(F)c2)cc(C)n1	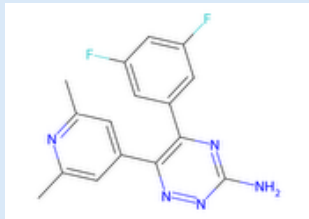	-9.98 ± 0.92	-11.64 ± 0.92	-10.31
**4j**	Cc1cc(-c2nnc(N)nc2-c2cccc(F)c2)cc(C)n1	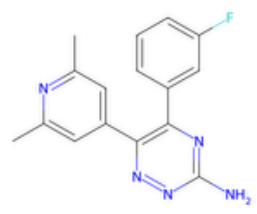	-10.5 ± 1.73	-11.42 ± 1.73	-10.88
**4k**	Cc1cc(-c2nnc(N)nc2-c2ccccc2)cc(C(F)(F)F)n1	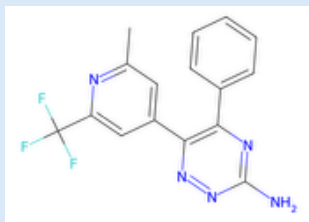	-12.67 ± 0.39	-12.77 ± 0.39	-11.53
**4l**	Cc1cc(-c2nnc(N)nc2-c2ccc(F)cc2)cc(C(F)(F)F)n1	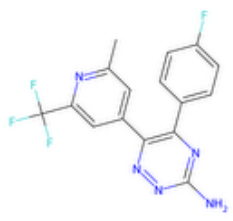	-10.24 ± 1.21	-10.75 ± 1.21	-11.37
**4m**	Nc1nnc(-c2ccnc(Cl)c2)c(-c2ccccc2)n1	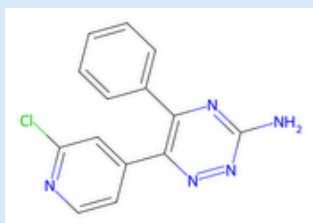	-9.99 ± 0.66	-11.37 ± 0.66	-10.5
**4n**	Nc1nnc(-c2cc(Cl)nc(Cl)c2)c(-c2ccccc2)n1	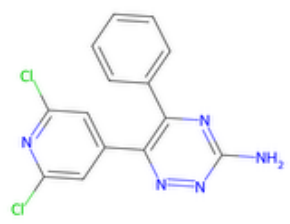	-12.18 ± 0.70	-12.55 ± 0.70	-11.59
**4q**	Nc1nnc(-c2ccncc2)c(-c2ccccc2)n1	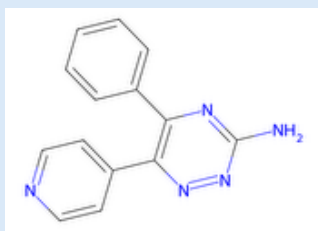	-7.83 ± 1.06	-9.44 ± 1.06	-8.86
**4r**	Nc1nnc(-c2cc(Cl)nc(C(F)(F)F)c2)c(-c2ccc(F)cc2)n1	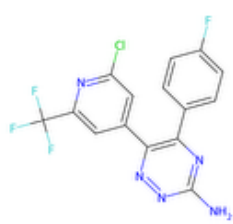	-12.22 ± 0.74	-14.26 ± 0.74	-11.45

Table S7. Ligand-specific comparison of global and compound-wise corrected absolute binding free energy predictions for the Deflorian et al. dataset in the presence of Na+. Canonical isomeric SMILES, ligand structures, corrected predicted binding free energies, SD, and experimental binding free energies are reported in kcal mol−1.

ligand	min_overlap_no_GCMC	min_overlap_with_GCMC	delta_min	median_overlap_no_GCMC	median_overlap_with_GCMC	delta_median
**6**	0.06	0.06	0	0.12	0.11	-0.01
**7**	0.03	0.03	0	0.1	0.11	0.01
**8**	0.03	0.03	0	0.14	0.12	-0.02
**9**	0.04	0.05	0.01	0.12	0.11	-0.01
**10**	0.05	0.05	0	0.11	0.12	0.01
**11**	0.04	0.04	0	0.11	0.15	0.04
**12**	0.06	0.03	-0.03	0.12	0.11	-0.01
**13**	0.04	0.03	-0.01	0.12	0.11	-0.01
**14**	0.04	0.04	0	0.13	0.12	-0.01
**15**	0.03	0.04	0.01	0.12	0.11	-0.01
**16**	0.03	0.03	0	0.15	0.12	-0.03
**17**	0.05	0.04	-0.01	0.13	0.15	0.02

Table S8. Adjacent-state overlap diagnostics for the mGlu5 complex-leg alchemical calculations, run with and without the GCMC resolvation procedure. For each ligand we report the minimum and median of the nearest-neighbour elements of the MBAR overlap matrix ($O_{i,i+1}$); Δ is with-GCMC minus no-GCMC. Both statistics change only marginally, indicating that resolvation did not materially alter phase-space overlap along the alchemical path. Absolute overlap remains above 0.03 in every window under both protocols, so the residual errors in the computed free energies are unlikely to originate in poor overlap between intermediate states.

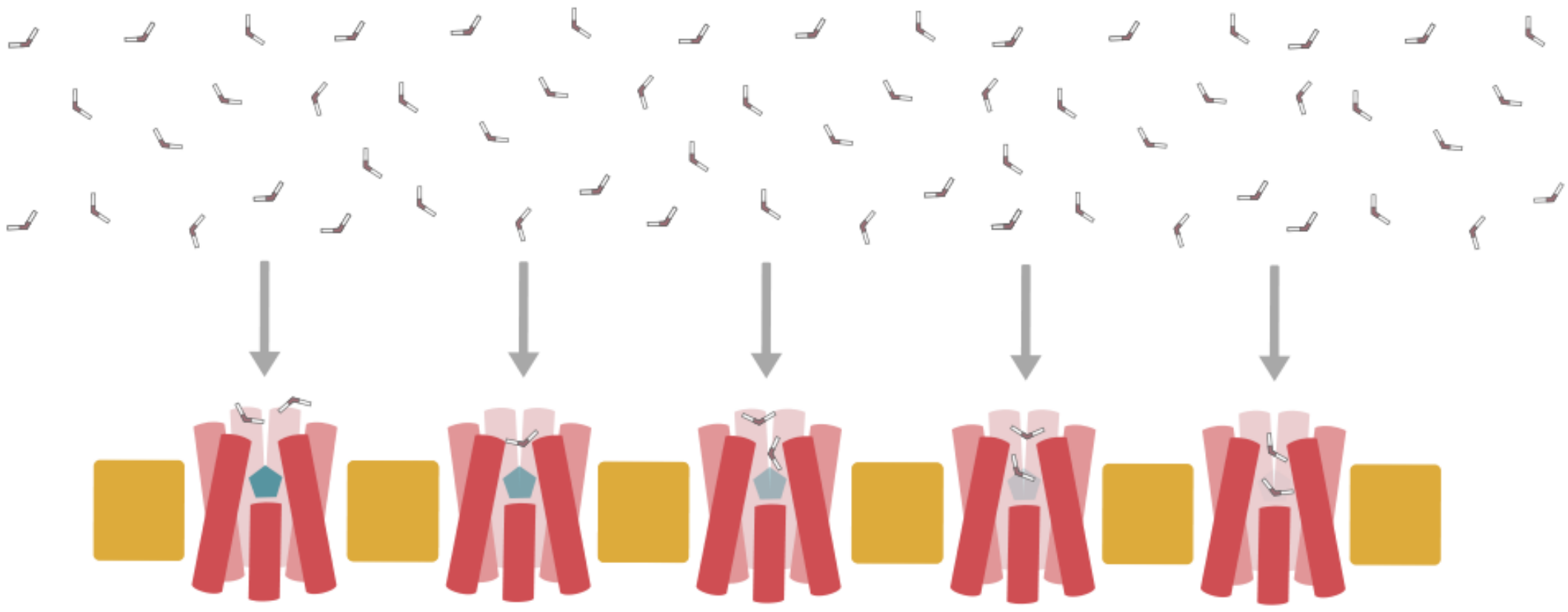

Figure S1. Schematic illustrating the GCMC-like process used to resolvate the protein binding pocket in ABFE windows approaching complete ligand annihilation. When the binding pocket is occupied by an interacting ligand (left), fewer water molecules enter the pocket. In contrast, when the ligand is fully decoupled (right), more water molecules are inserted into the pocket via GCMC like solvation.

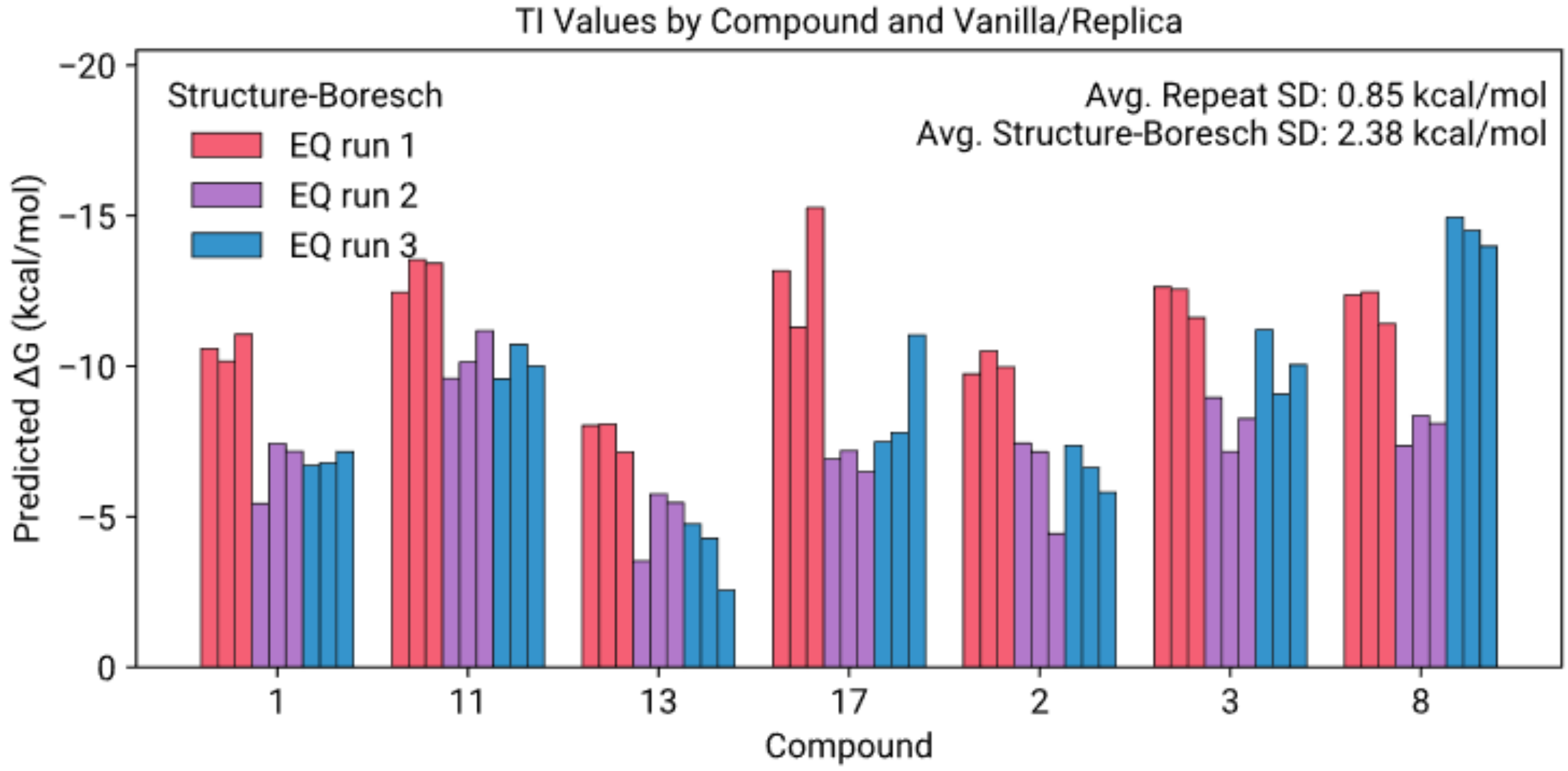

Figure S2. Predicted absolute binding free energies (ABFEs) for the Lenselink *et al*. dataset. Individual bars represent affinity estimates from repeated ABFE calculations, colored by Boresch restraint–structure setup. Across compounds, variability between different Boresch–structure setups exceeds the variability between repeats within the same setup, indicating that the choice of Boresch restraint and starting structure is a dominant source of ABFE variability.

a.

b.

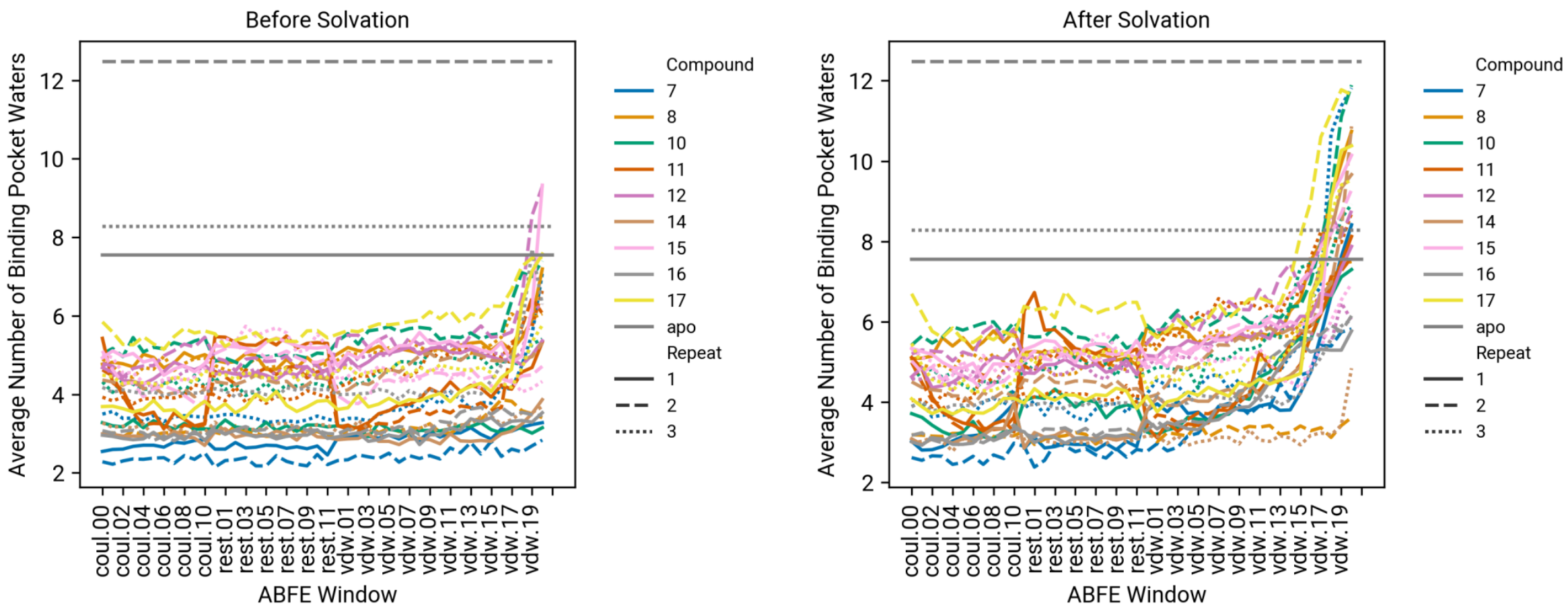

Figure S3. Water-occupancy plots across ABFE windows for additional compounds not shown in Figure 4 of the main manuscript. (a) Before and (b) after the solvation step. Most compounds reach apo-level solvation in the final van der Waals decoupling leg after applying the solvation scheme, demonstrating its effectiveness in introducing water into the binding pocket.

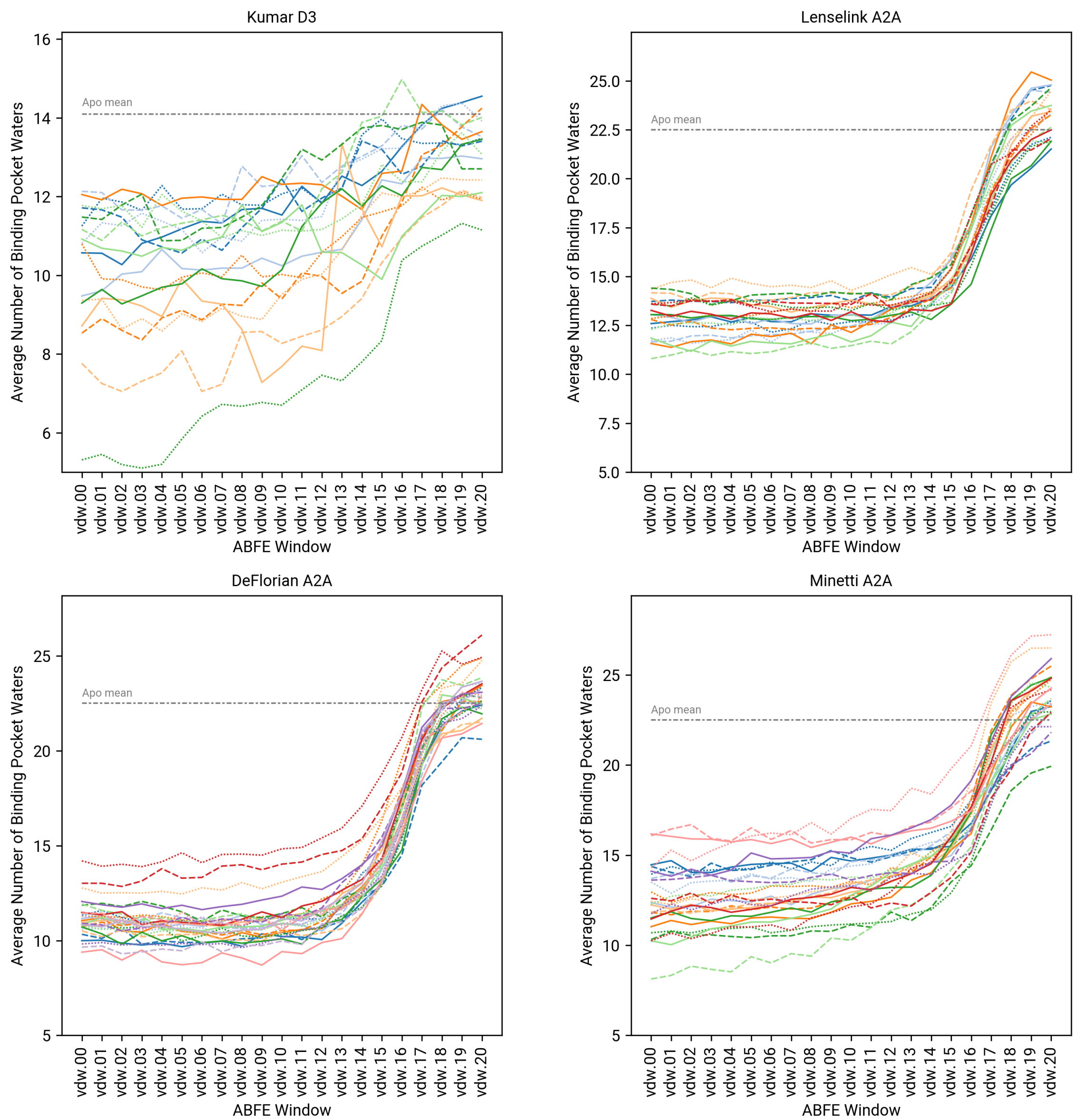

Figure S4. Binding-pocket water occupancy across the VDW decoupling windows for the Kumar D3, Lenselink A2A, DeFlorian A2A, and Minetti A2A datasets. Colored traces denote individual ligands, with line style indicating independent ABFE repeats. The grey horizontal line shows the mean apo binding-pocket water occupancy for each dataset. These plots compare ligand-decoupled pocket resolvation against the corresponding apo-state reference.

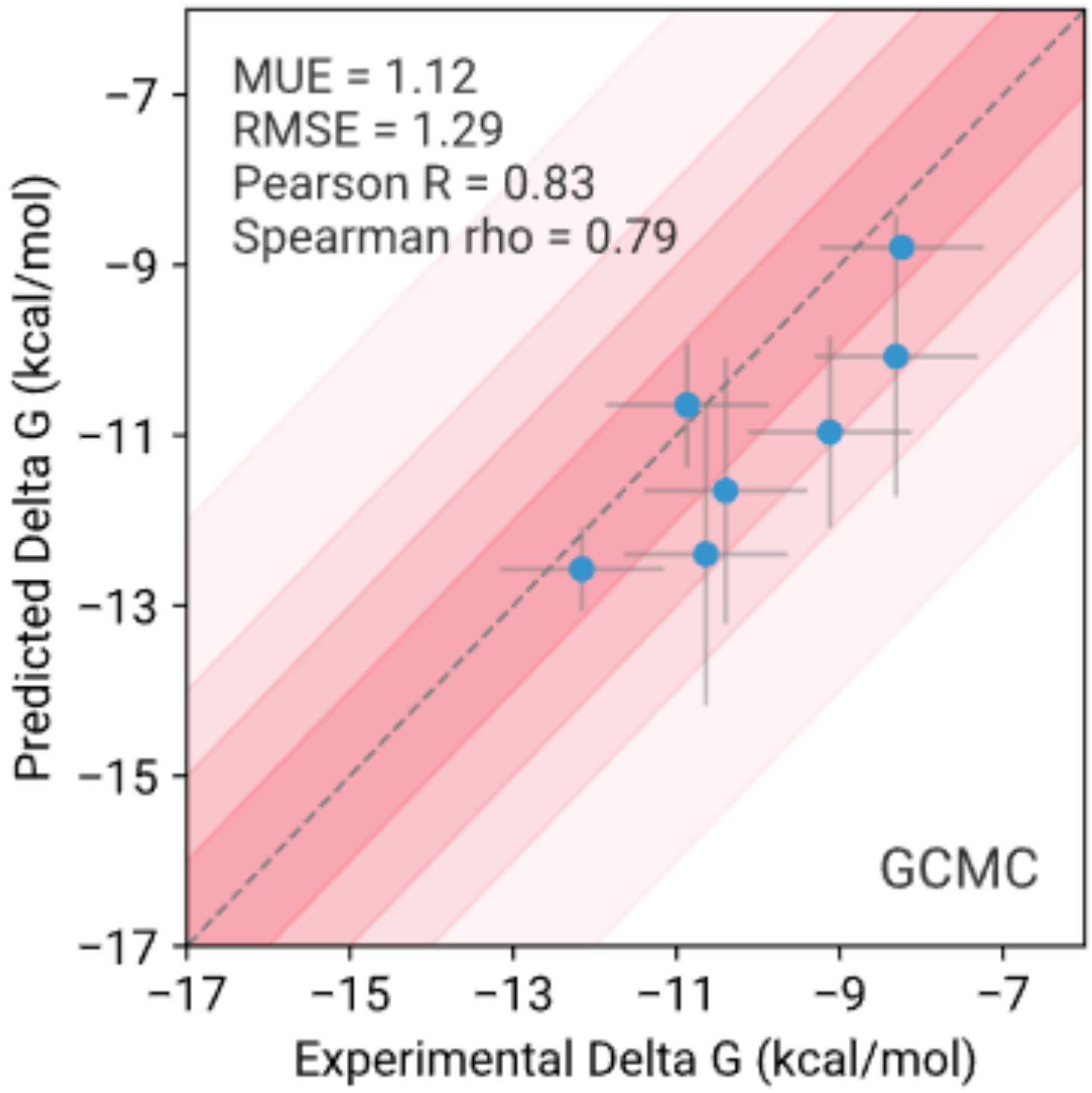

Figure S5. Experimental versus predicted binding free energies for Lenselink et al dataset with GCMC like resolvation. Error bars show the standard deviation across starts. Shaded diagonal bands indicate deviations of 1, 2, 3, and 5 kcal/mol from perfect agreement.

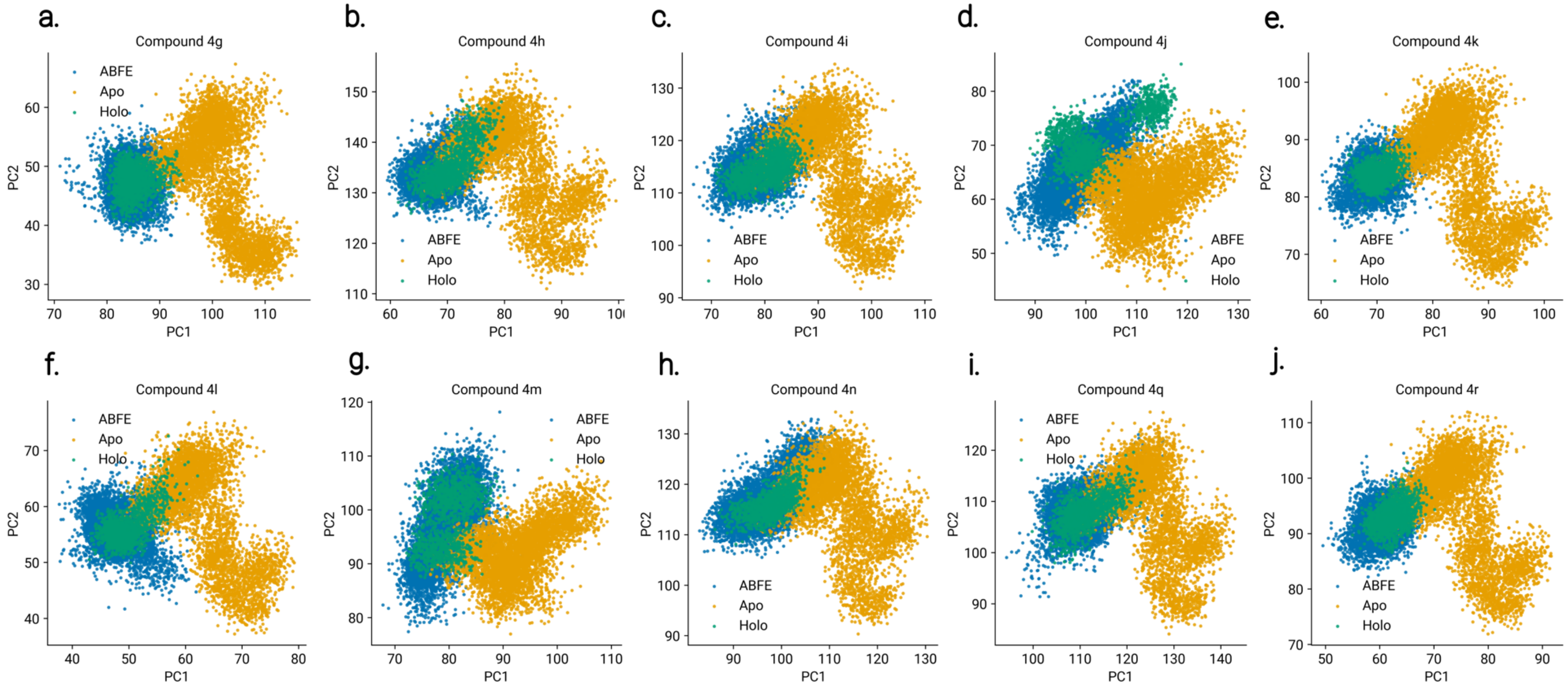

Figure S6. PCA plots of pairwise Cα distance features colored by simulation type for (a) 4g, (b) 4h, (c) 4i, (d) 4j, (e) 4k, (f) 4l (g) 4m (h) 4n, (i) 4q, and (j) 4r. The apo simulations follow a distribution that is distinct from both ABFE and holo distributions, while the ABFE distribution largely overlaps with the holo ensemble.

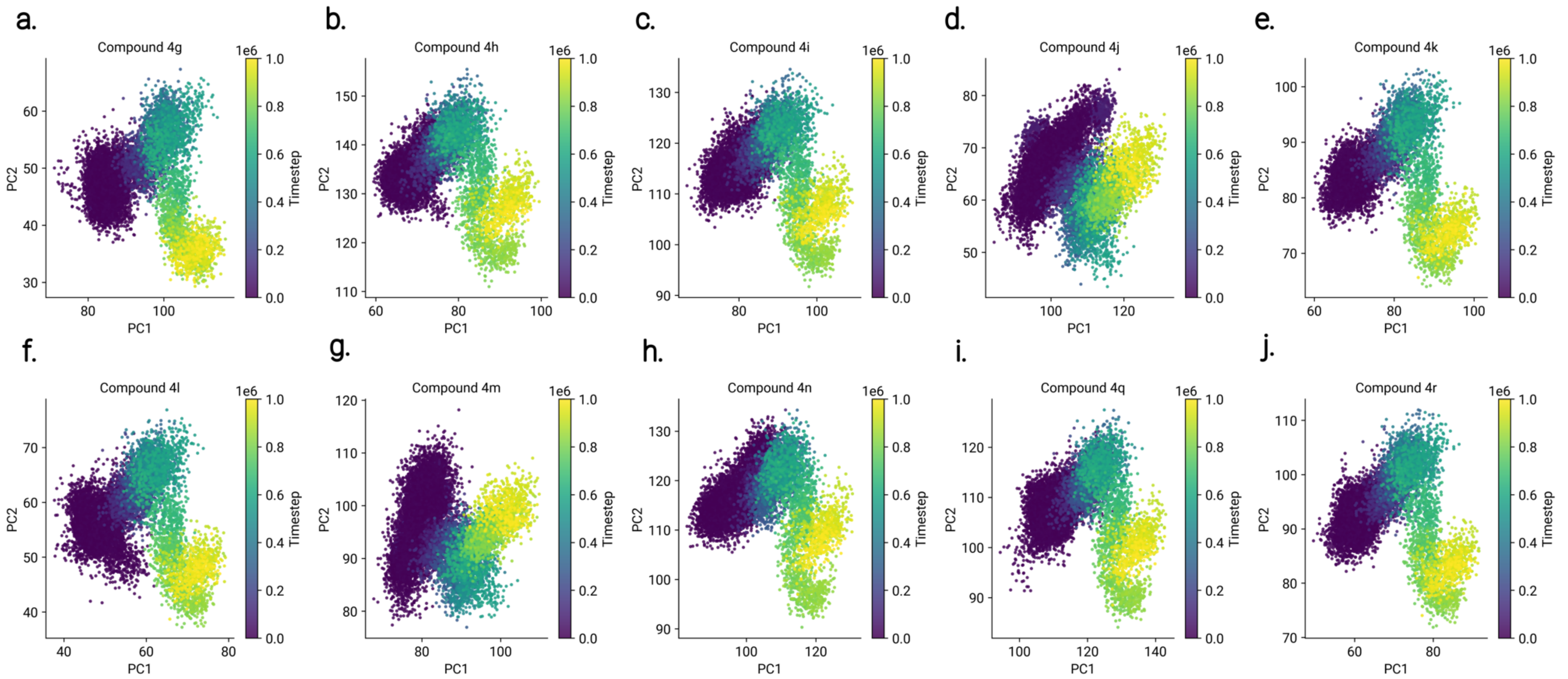

Figure S7. PCA plots of pairwise Cα distance features colored by simulation time for (a) 4g, (b) 4h, (c) 4i, (d) 4j, (e) 4k, (f) 4l (g) 4m (h) 4n, (i) 4q, and (j) 4r. The apo simulations form a distinct cluster from ABFE and holo states. In addition, earlier apo trajectories tend to concentrate near the center of the apo distribution.

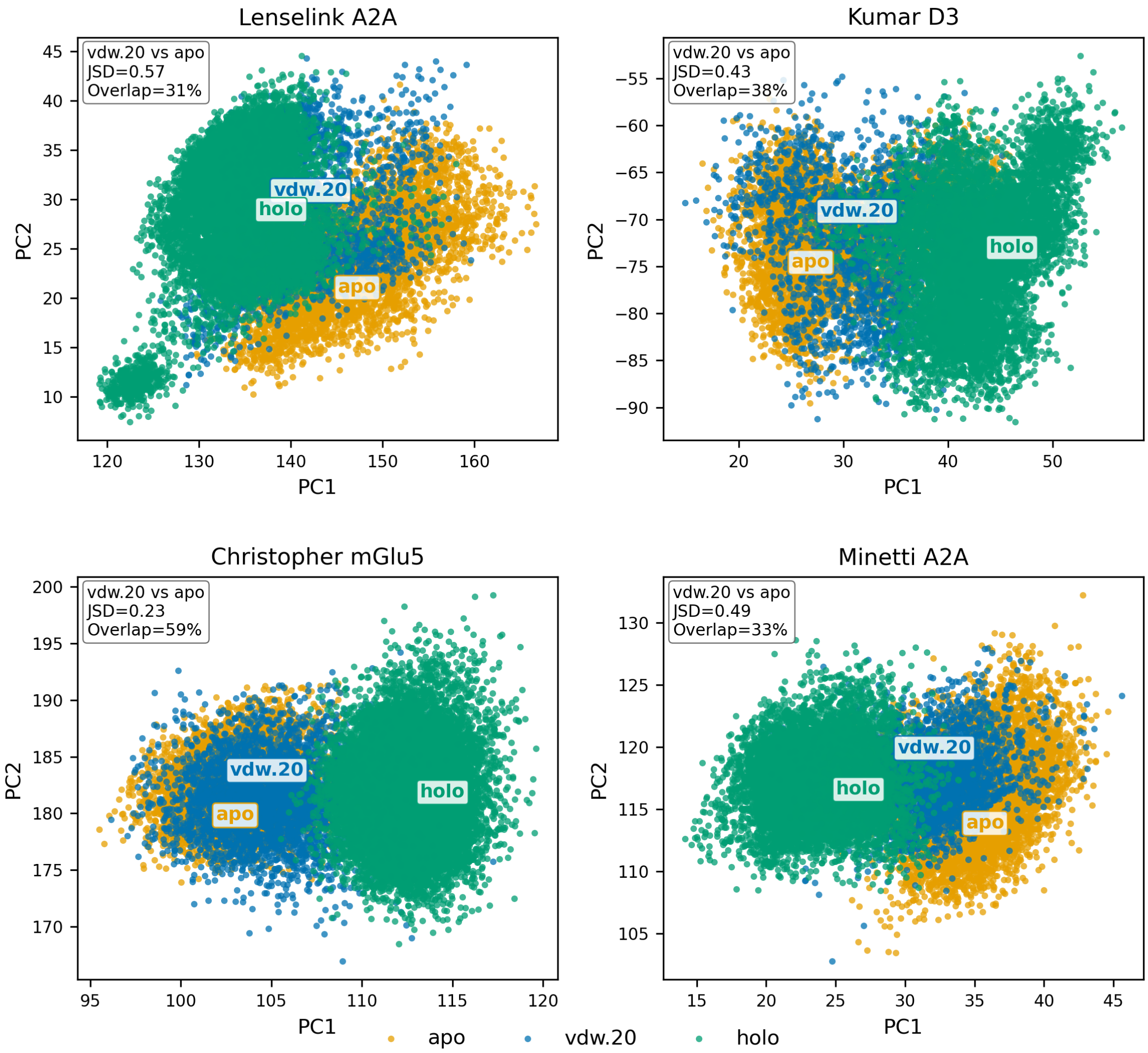

Figure S8. Binding-pocket PCA comparison of apo, terminal alchemical vdw.20, and holo ensembles across the Lenselink A2A, Kumar D3, Christopher mGlu5, and Minetti A2A datasets. PCA projections were generated independently for each dataset from pooled ligand binding-pocket pairwise distance features. Points represent trajectory frames projected onto PC1 and PC2; colors denote apo, vdw.20, and holo ensembles. Insets report Jensen-Shannon divergence (JSD) and histogram overlap between vdw.20 and the apo density remaining after removing holo-overlapping apo density, with lower JSD and higher overlap indicating greater similarity to the apo region adjusted for initial equilibration.

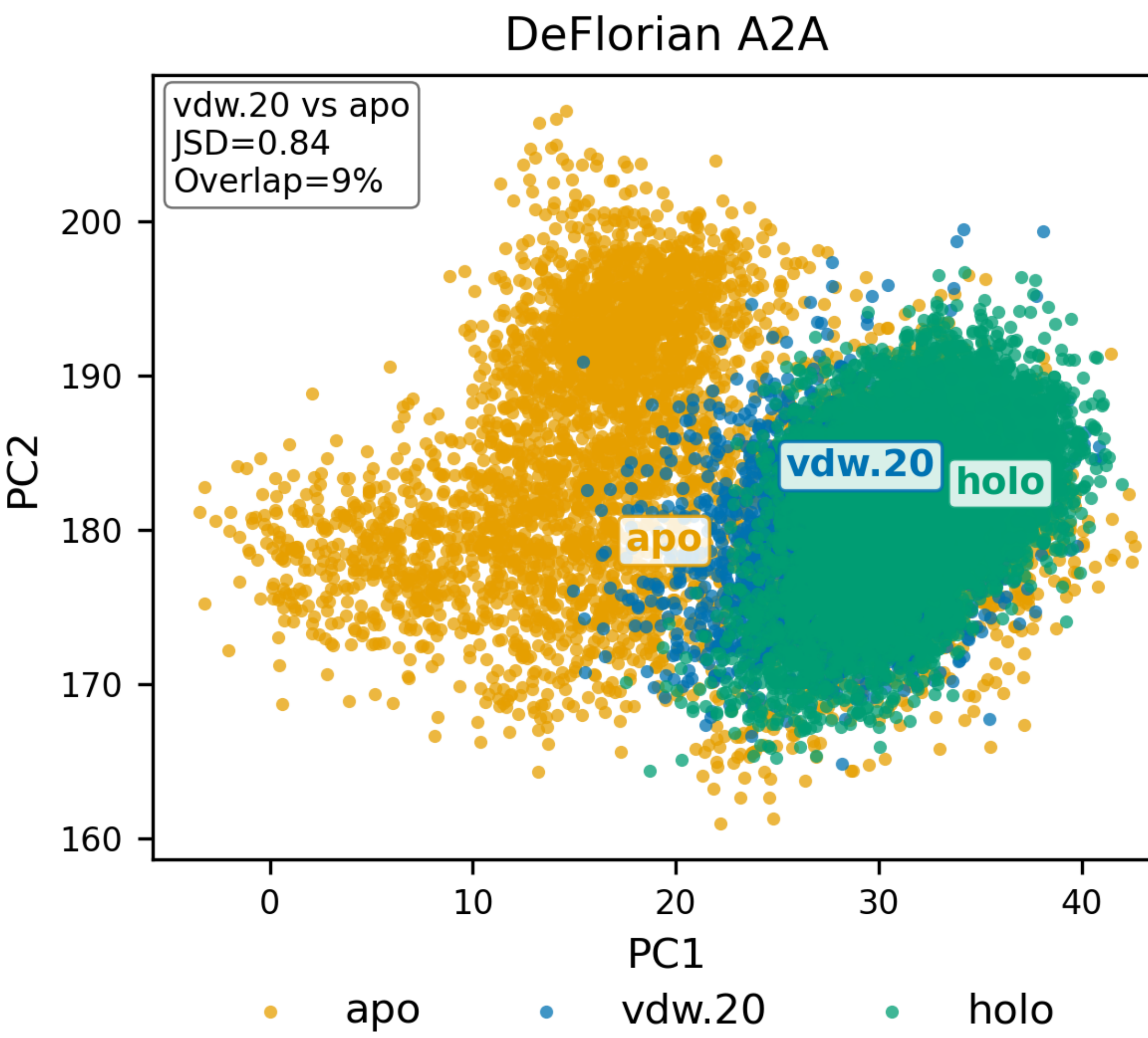

Figure S9. Binding-pocket PCA comparison of apo, terminal alchemical vdw.20, and holo ensembles for the Deflorian A2A dataset. Points represent trajectory frames projected onto PC1 and PC2 from pooled ligand binding-pocket pairwise distance features. Colors denote apo, vdw.20, and holo ensembles. The inset reports JSD and histogram overlap between vdw.20 and the apo density remaining after subtracting holo-overlapping apo density, quantifying the extent to which the alchemical endpoint samples the equilibrated apo region.

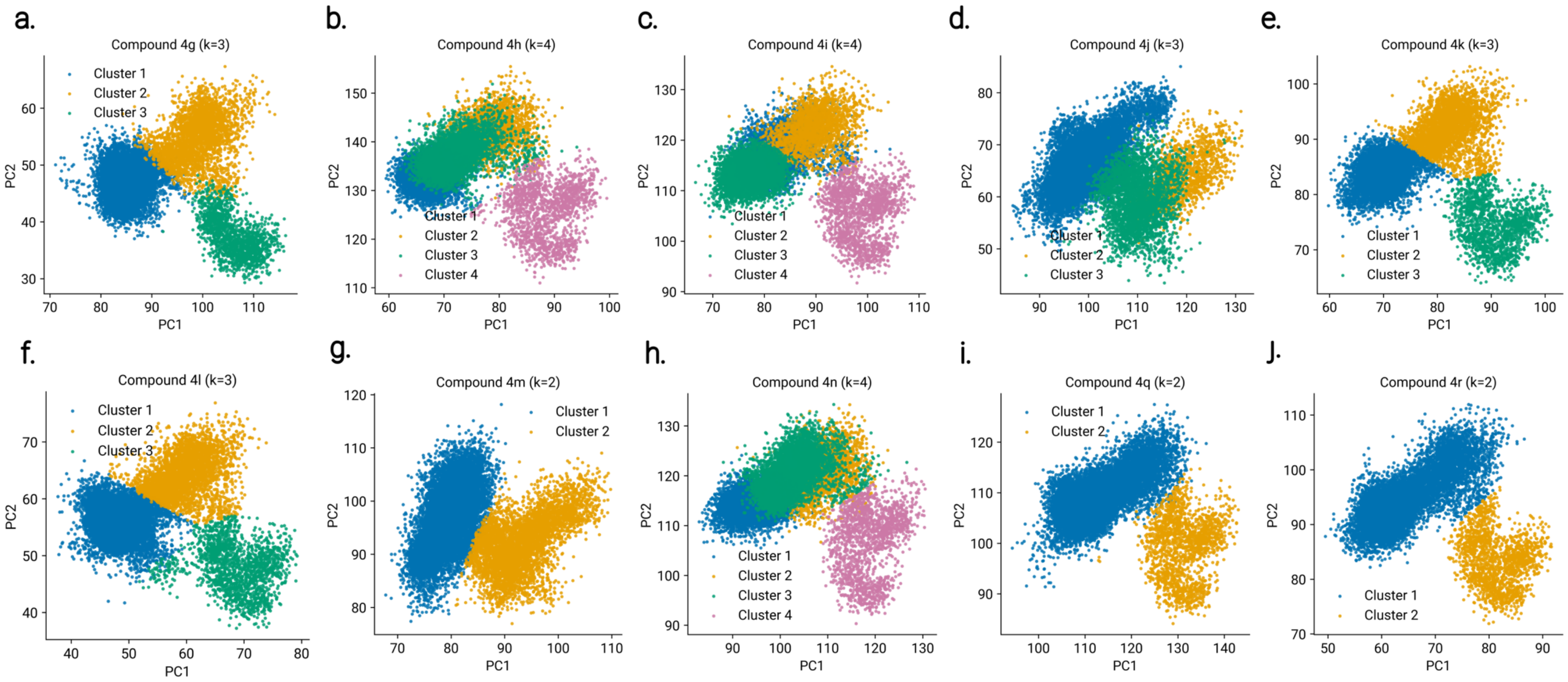

Figure S10. PCA plots of pairwise Cα distance features after clustering, colored by cluster assignment for (a) 4g, (b) 4h, (c) 4i, (d) 4j, (e) 4k, (f) 4l (g) 4m (h) 4n, (i) 4q, and (j) 4r. The number of clusters identified vary from two primary clusters corresponding to apo and holo states to up to four distinct clusters. In cases where there are more than one ABFE clusters, the cluster with the highest proportion of apo structures was selected as the apo cluster for subsequent REUS simulations. The clusters selected as the apo cluster for each compound are as follows: 4g: 3, 4h: 4, 4i: 4, 4j: 3, 4k: 3, 4l: 3, 4m: 2, 4n: 4, 4q: 2, and 4r: 2.

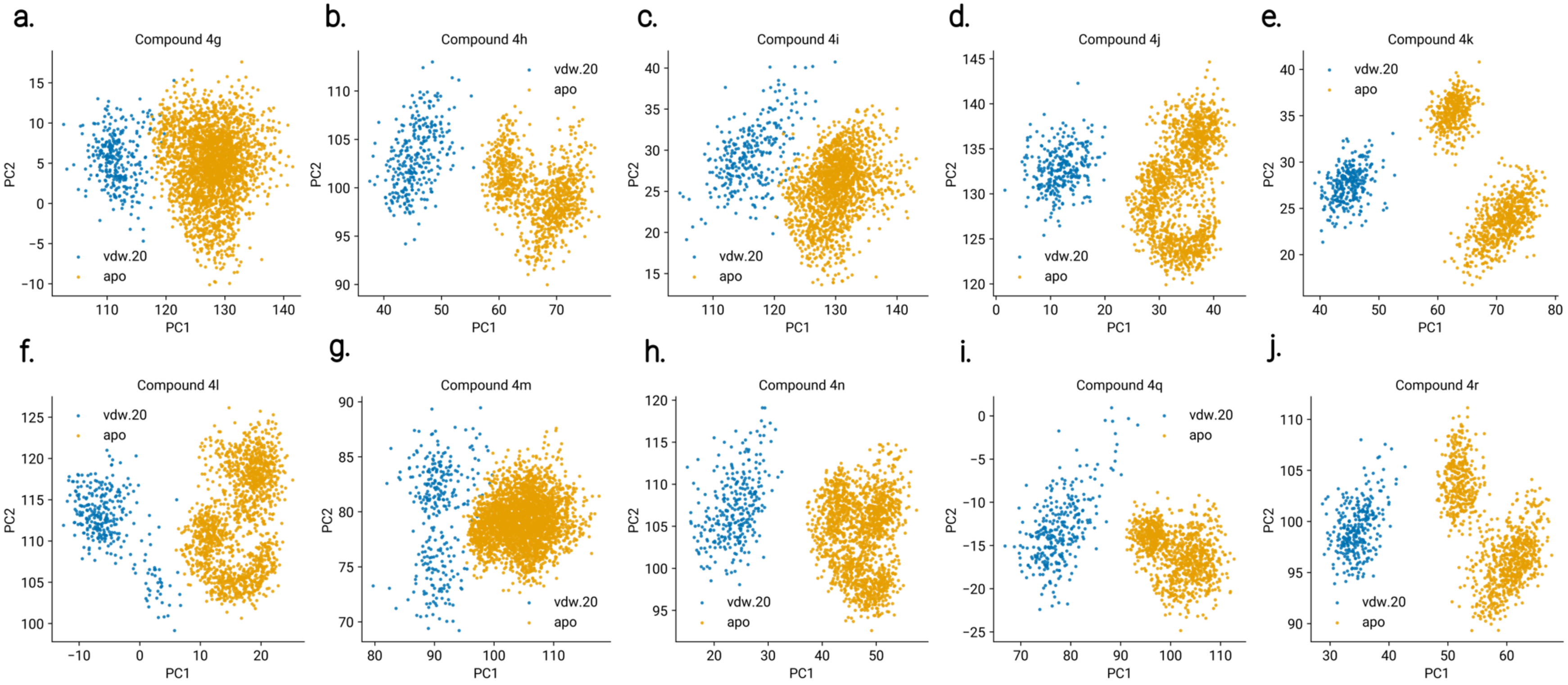

Figure S11. PCA plots of a subset of simulations (vdw.20) together with the apo cluster identified in Figure S10 for (a) 4g, (b) 4h, (c) 4i, (d) 4j, (e) 4k, (f) 4l (g) 4m (h) 4n, (i) 4q, and (j) 4r. The apo conformations form a distinct cluster from both ABFE and vdw.20 simulations. The first principal component (PC1) from these projections is used as the collective variable for the REUS simulations.

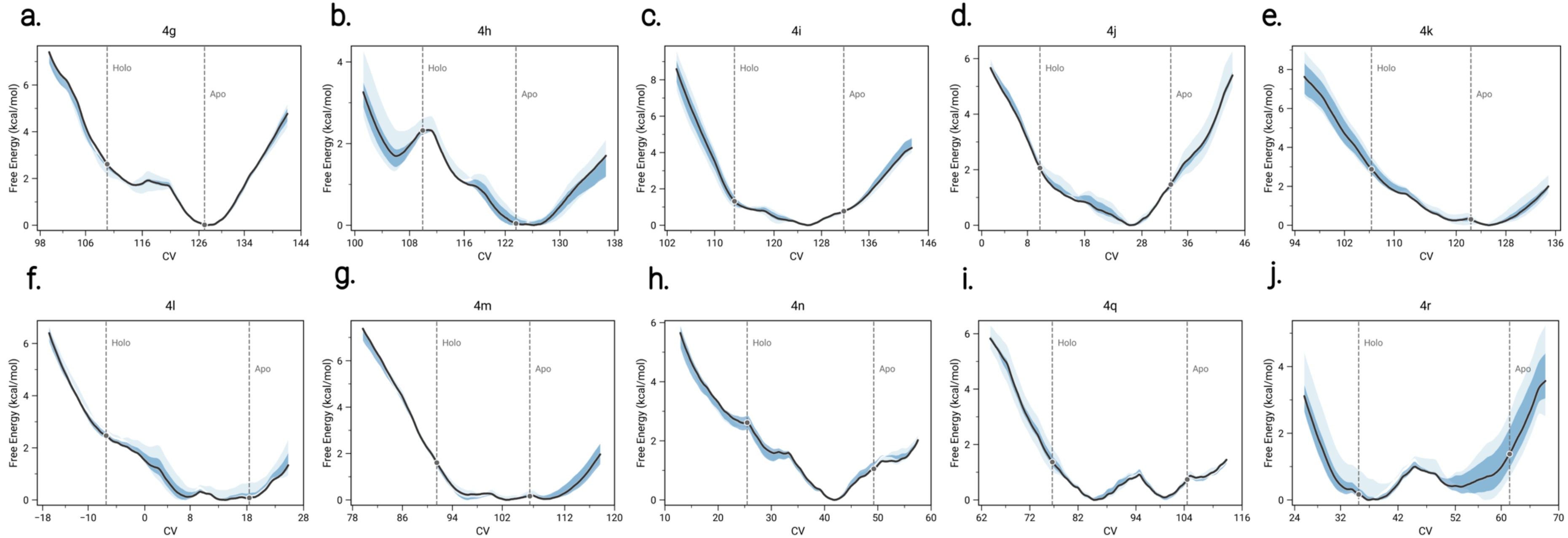

Figure S12. Free energy surfaces (FES) obtained from replica exchange umbrella sampling (REUS) simulations for compounds (a) 4g, (b) 4h, (c) 4i, (d) 4j, (e) 4k, (f) 4l (g) 4m (h) 4n, (i) 4q, and (j) 4r. Compounds 4g and 4q exhibit distinct holo wells, whereas the remaining compounds display a single apo well.

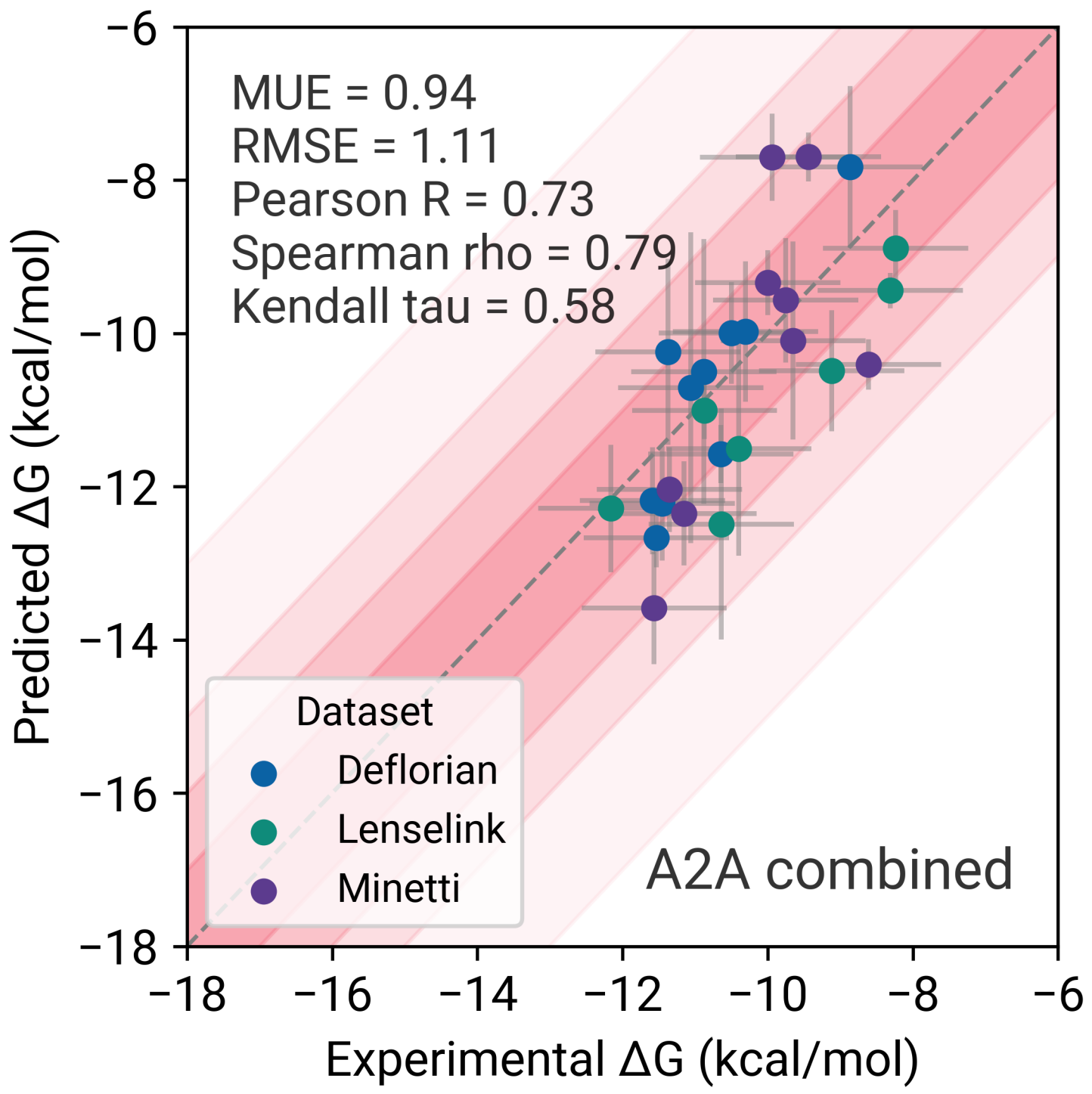

Figure S13. Combined ABFE performance for the A2A adenosine receptor across ligand series. Predicted binding free energies are plotted against experimental values for the pooled Deflorian et al, Lenselink et al, and Minetti et al A2A antagonist datasets (n = 26), colored by source dataset (blue, teal, purple, respectively). The dashed line marks exact agreement; shaded bands denote ± 1, ± 2, ± 3, and ± 5 kcal/mol deviations. Error bars reflect experimental and predicted uncertainties. Performance metrics (MUE, RMSE, Pearson R, Spearman ρ, Kendall τ) are computed over the pooled dataset.

References

(1) Doré, A. S.; Okrasa, K.; Patel, J. C.; Serrano-Vega, M.; Bennett, K.; Cooke, R. M.; Errey, J. C.; Jazayeri, A.; Khan, S.; Tehan, B.; et al. Structure of class C GPCR metabotropic glutamate receptor 5 transmembrane domain. *Nature* **2014**, *511* (7511), 557-562. DOI: 10.1038/nature13396. Koehl, A.; Hu, H.; Feng, D.; Sun, B.; Zhang, Y.; Robertson, M. J.; Chu, M.; Kobilka, T. S.; Laeremans, T.; Steyaert, J.; et al. Structural insights into the activation of metabotropic glutamate receptors. *Nature* **2019**, *566* (7742), 79-84. DOI: 10.1038/s41586-019-0881-4.

(2) Michino, M.; Free, R. B.; Doyle, T. B.; Sibley, D. R.; Shi, L. Structural basis for Na+-sensitivity in dopamine D2 and D3 receptors. *Chemical Communications* **2015**, *51* (41), 8618-8621, 10.1039/C5CC02204E. DOI: 10.1039/C5CC02204E. Newton, C. L.; Wood, M. D.; Strange, P. G. Examining the Effects of Sodium Ions on the Binding of Antagonists to Dopamine D2 and D3 Receptors. *PLOS ONE* **2016**, *11* (7), e0158808. DOI: 10.1371/journal.pone.0158808. Xu, P.; Huang, S.; Mao, C.; Krumm, B. E.; Zhou, X. E.; Tan, Y.; Huang, X.-P.; Liu, Y.; Shen, D.-D.; Jiang, Y.; et al. Structures of the human dopamine D3 receptor-Gi complexes. *Molecular Cell* **2021**, *81* (6), 1147-1159.e1144. DOI: https://doi.org/10.1016/j.molcel.2021.01.003.

(3) Gutiérrez-de-Terán, H.; Massink, A.; Rodríguez, D.; Liu, W.; Han, Gye W.; Joseph, Jeremiah S.; Katritch, I.; Heitman, Laura H.; Xia, L.; Ijzerman, Adriaan P.; et al. The Role of a Sodium Ion Binding Site in the Allosteric Modulation of the A_{2A} Adenosine G Protein-Coupled Receptor. *Structure* **2013**, *21* (12), 2175-2185. DOI: 10.1016/j.str.2013.09.020 (acccessed 2026/06/01). Katritch, V.; Fenalti, G.; Abola, E. E.; Roth, B. L.; Cherezov, V.; Stevens, R. C. Allosteric sodium in class A GPCR signaling. *Trends in Biochemical Sciences* **2014**, *39* (5), 233-244. DOI: https://doi.org/10.1016/j.tibs.2014.03.002. Liu, W.; Chun, E.; Thompson, A. A.; Chubukov, P.; Xu, F.; Katritch, V.; Han, G. W.; Roth, C. B.; Heitman, L. H.; Ijzerman, A. P.; et al. Structural Basis for Allosteric Regulation of GPCRs by Sodium Ions. *Science* **2012**, *337* (6091), 232-236. DOI: 10.1126/science.1219218 (acccessed 2026/04/17). Ye, L.; Neale, C.; Sljoka, A.; Lyda, B.; Pichugin, D.; Tsuchimura, N.; Larda, S. T.; Pomès, R.; García, A. E.; Ernst, O. P.; et al. Mechanistic insights into allosteric regulation of the A2A adenosine G protein-coupled receptor by physiological cations. *Nature Communications* **2018**, *9* (1), 1372. DOI: 10.1038/s41467-018-03314-9.
